# Discovery of a photoactivatable dimerized STING agonist based on the benzo[*b*]selenophene scaffold†

**DOI:** 10.1039/d2sc06860e

**Published:** 2023-03-13

**Authors:** Dongyu Liu, Bin Yu, Xin Guan, Bin Song, Huikai Pan, Renbing Wang, Xi Feng, Lixia Pan, Huidan Huang, Zhe Wang, Hongxi Wu, Zhixia Qiu, Zhiyu Li, Jinlei Bian

**Affiliations:** a Department of Medicinal Chemistry, School of Pharmacy, China Pharmaceutical University Nanjing 211100 P. R. China bianjl@cpu.edu.cn; b State Key Laboratory of Non-Food Biomass and Enzyme Technology, Guangxi Academy of Sciences Nanning 530007 P. R. China; c Department of Pharmaceutical Engineering, School of Pharmacy, Wannan Medical College Wuhu China

## Abstract

Stimulator of interferon genes (STING) agonism presents a powerful weapon for cancer immunotherapy. This study reports a novel dimerized STING agonist diBSP01, which exhibited promising STING binding and activation properties *in vitro*, based on the benzo[*b*]selenophene scaffold. Meanwhile, shielding the pharmacophores of diBSP01 with photoremovable protecting groups (PPGs) resulted in the generation of the first photoactivatable STING agonist, caged-diBSP01, that exerted no biological potency in the absence of light stimulation while regaining its STING agonistic activity after 400 nm irradiation. Optically controlled *in vivo* anticancer activity was also proven with caged-diBSP01 in a zebrafish xenograft model. Our study provides insights into developing novel STING agonists for cancer treatment and a solution for precise STING activation to avoid the on-target systemic inflammatory response responsible for normal cell damage caused by systemic STING agonism.

## Abstract 2

The first photoactivatable STING agonist, caged-diBSP01, is presented based on a novel and potent dimerized STING agonist diBSP01. Caged-diBSP01 allowed spatiotemporal control of STING activation by light, which was well characterized both *in vitro* and *in vivo*.

## Graphical abstract

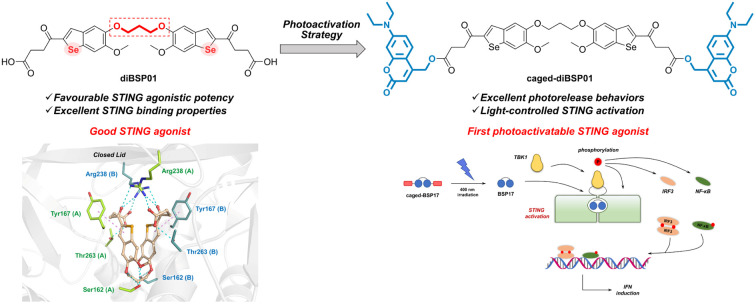

The cyclic GMP-AMP synthase (cGAS)–stimulator of interferon genes (STING) pathway is one of the most vital components in initiating innate immune responses upon endogenous (dead and tumor cells) or exogenous (bacterial and viral infections) stimulation.^1^ Cytosolic aberrant double-stranded DNA (dsDNA) is detected by cGAS, followed by the generation of cyclic GMP-AMP (cGAMP) that serves as a second messenger to bind to and activate STING, after which the endoplasmic reticulum (ER) membrane-anchored STING translocates to perinuclear compartments such as the Golgi apparatus.^2,3^ STING then recruits and promotes the autophosphorylation of TANK-binding kinase 1 (TBK1) at the C-terminal tail and the phosphorylated TBK1 in turn phosphorylates STING tails that further recruit and deliver transcription factor interferon regulatory factor 3 (IRF3) to TBK1 for its phosphorylation.^4–6^ In addition, TBK1 recruitment is also of great importance, activating transcription factor nuclear factor κB (NF-κB).^7^ The activated IRF3 dimer and NF-κB together enter the nucleus and act synergistically to promote the production of type I interferons (IFNs) and other proinflammatory factors (TNFα, IL6, and CXCL10), which ultimately initiates innate immune system and subsequent adaptive immune responses.^8,9^

In particular, the ER membrane protein STING has been regarded as crucial in regulating such effects and has received increasing attention from scientists not only to shed light on its physiological functions but also to develop modulators targeting the STING adaptor for various diseases.^10–12^ Indeed, given the potential benefits of STING activation in tumor immunotherapy, numerous companies and institutes have dedicated themselves to discovering STING agonists to treat cancer. Most of the agents currently in clinical trials are first-generation STING agonists, which are cyclic dinucleotide (CDN) derivatives that mimic the natural ligand cGAMP. These agents have their own deficiencies, including high molecular weight, poor membrane permeability, and poor stability, which impede their further application.^13^ The discovery of next-generation STING agonists with improved druggability and pharmacokinetic/pharmacodynamic profiles has led to several promising non-CDN agonists, represented by diABZI-Compound 3 from GSK,^14^SR717 from Scripps Research,^15^ and MSA-2 from Merck,^16^ all of which exhibit encouraging efficacy *in vitro* and *in vivo*. Moreover, several impressive studies of the structure–activity relationships of these molecules have been reported in succession.^17–20^ Developing activators targeting STING has provided an impetus to cancer immunotherapy, and STING agonists with novel chemical entities are urgently required for propelling their application. However, the systemic administration of STING agonists is likely to induce a robust and inevitable on-target ‘cytokine storm’ that may cause damage to normal tissues, limiting the further clinical use of these agents.^21,22^

Recently, the emerging role of the photoactivation strategy has provided a solution to this challenge. Upon pharmacological inactivation of a small molecule by concealing a functional group with a photoremovable protecting group (PPG), temporal and spatial control can be accomplished after irradiation with ultraviolet (UV)-visible light.^23,24^ Given the on-demand activation properties, multitudes of pharmacological agents disguised with various types of PPGs have been designed for precise treatment.^25–28^ Despite the extensive applications of this strategy in prodrug design, no photoactivatable STING agonists have been reported yet.

This study describes the discovery of a novel dimerized STING agonist diBSP01, which exhibited promising binding properties and agonistic potency *in vitro*, based on the benzo[*b*]selenophene scaffold. In addition, a photo-sensitive STING agonist is presented for the first time by using a coumarin-based PPG on carboxylic acid moieties representing the critical pharmacophores for STING binding. As expected, caged-diBSP01 was incapable of activating the STING pathway, while light irradiation resulted in the efficient cleavage of the PPG moiety and liberation of the parent drug responsible for the restored agonistic activity. In addition, the light-controllable *in vivo* anticancer activity of caged-diBSP01 was well characterized.

Our molecular design was initiated with a recently disclosed STING agonist, MSA-2 (Fig. 1A), identified by Merck.^16^MSA-2 is the first orally available non-nucleotide STING agonist having tremendous potency *in vivo* in various mouse syngeneic tumor models. It has a relatively simple yet ingenious chemical structure, which indicates the presence of abundant space for chemical modification. Selenium (Se) is a chalcogen participating in a wide range of physiological events. As a favorable bioisostere related to oxygen and sulfur (S), Se-containing compounds are appreciated not only for their improved physicochemical profiles but also for their remarkable anti-inflammatory, antitumoral, antibacterial, and/or antiviral properties.^29–31^ In addition, Se is capable of activating immune cells, resulting in the reversal of immunosuppression in the tumor microenvironment, and promoting the secretion of proinflammatory cytokines such as IFN-γ.^32^ Considering the promising prospects of incorporating Se into bioactive molecules and the potential antitumor immune responses of Se compounds, a bioisostere strategy was implemented to replace the S in MSA-2 with Se so as to exert the potential synergistic antitumor effect of Se and STING activation, which resulted in the generation of a novel compound with the benzo[*b*]selenophene scaffold, namely BSP01 (Fig. 1A, see the ESI† for the synthesis). However, this simple modification of BSP01 resulted in no binding affinity towards hSTING^H232^ protein in a differential scanning fluorimetry (DSF) assay (Fig. S1A†) and a dramatically decreased activity in an interferon-stimulated response element (ISRE) luciferase reporter assay in both human-derived cells (ISG-THP1 cells) and murine-derived cells (ISG-RAW264.7 cells) (Fig. S1B†), suggesting that BSP01 is a weak STING agonist. The underlying mysteries emerging from these results remain unclear; however, we speculated that the incorporation of selenium might increase the electron cloud radius of the benzoselenophene ring, leading to a change in the dihedral angle of C2/C3 from the butyric acid side chain (Fig. S2†) and thus an improper orientation of the terminal carboxyl in the binding pocket and weak interactions with Arg238, which is a crucial residue for STING binding and activation.^16^

**Fig. 1:**
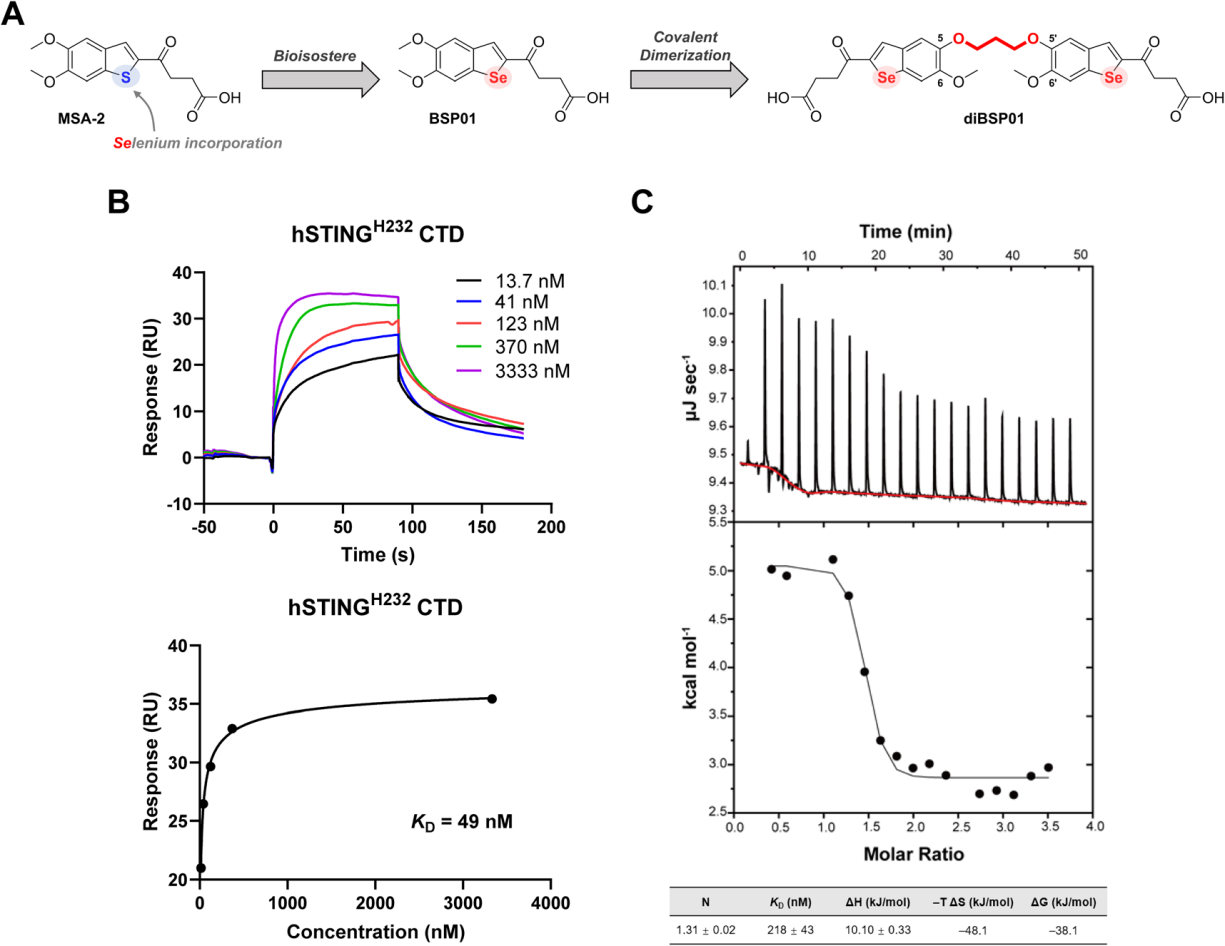
Discovery of a novel dimerized STING agonist diBSP01 and evaluation of its binding ability. (A) Design strategy of BSP01 and diBSP01. (B) Concentration-dependent SPR binding of diBSP01 to immobilized hSTING^H232^ (above) and corresponding Langmuir-binding isotherm (below). (C) Validation of the interaction between diBSP01 and hSTING^H232^ by ITC at 25 °C. Experiments were performed three times and values are presented as mean ± standard deviation (SD).

MSA-2 can preorganize in solution to form a bioactive noncovalent dimer, and then enter the ligand-binding pocket of STING and exert its pharmacological effects. Hence, Merck's researchers expended considerable efforts in searching for more potent covalent dimer STING agonists.^16^ Dimer 3 that tethers benzothiophene between 5-positions with an all-carbon propyl linker was finally selected as a surrogate of MSA-2 using a computational screening method based on the crystal structure of the MSA-2–STING complex combined with phenotypic experiments. In addition, these dimers had a broad accommodation for different linker compositions and positions, provided that the oxobutanoic acid side chains stretched properly in the binding pocket and that the carbonyl and carboxyl groups bonded correctly to the key amino acids.

Inspired by these observations, a dimerization strategy was implemented on BSP01 to restrict the conformation and reduce the entropy loss during the spontaneous dimerization process, which might ultimately increase the agonistic activity. Merck's alternative dimer 3 showed loss of interactions of the 5-methoxy groups with Ser162 because of its all-carbon linker between 5-positions,^16^ which was not desired because we wanted to preserve the original interactions as much as possible. Therefore, we revisited the co-crystal structure of the MSA-2–STING complex and extracted the information that the 5-O of one monomer and the 5′-O of the other monomer were adjacent to each other in the noncovalently dimerized MSA-2, with a distance of 3.1 Å (Fig. S3†). This implied that a three-carbon atom linker with a C–C bond length of 1.54 Å is a good choice for covalent connection of BSP01 between 5-O and 5′-O, which resulted in the formation of diBSP01 (Fig. 1A).

Following the synthesis of diBSP01 (see the ESI† for details), the thermostability of STING protein with diBSP01 was first evaluated. diBSP01 could increase the thermal denaturation temperature of hSTING^H232^ in a concentration-dependent manner (Fig. S4A†). In addition, diBSP01 also showed strong affinity towards hSTING^R232^ and hSTING^HAQ^ protein with Δ*T*_m_ = 16.2 °C and 27.0 °C, respectively (Fig. S4B and C†). These results indicated that diBSP01 restored its affinity to STING protein through dimerization. Surface plasmon resonance (SPR) and isothermal titration calorimetry (ITC) assays were then performed to confirm the binding ability of diBSP01 to STING. diBSP01 could induce the upregulation of the fluorescence signal in a concentration-dependent manner with a dissociation constant (*K*_D_) of 49 nM in SPR assay (Fig. 1B). Besides, in ITC assay, the binding of diBSP01 with STING effectively triggered a heat change and the binding ratio (*N*) was calculated to be 1.3 (Fig. 1C), suggesting that the stoichiometric ratio of the diBSP01–STING complex was 1 : 1. Moreover, the calculated *K*_D_ was 218 nM. Also, Δ*H* = 10.10 kJ mol^−1^, while −*T*Δ*S* = −48.1 kJ mol^−1^, which implied a binding mode driven by entropy (Fig. 1C).

The 2.7 Å co-crystal structure of diBSP01 bound to hSTING^H232^ was resolved to fully understand the interactions between diBSP01 and STING. As illustrated in Fig. 2A, diBSP01 was buried in the ligand-binding pocket as a monomeric molecule. The ligand adopted a U-like shape by folding the connecting linker leading to the parallel orientation of the two benzoselenophene rings with a distance of 3.6 Å between the two surfaces (Fig. 2A). Furthermore, the π–π stacking between the two rings was believed to be responsible for stabilizing this molecular conformation.

**Fig. 2:**
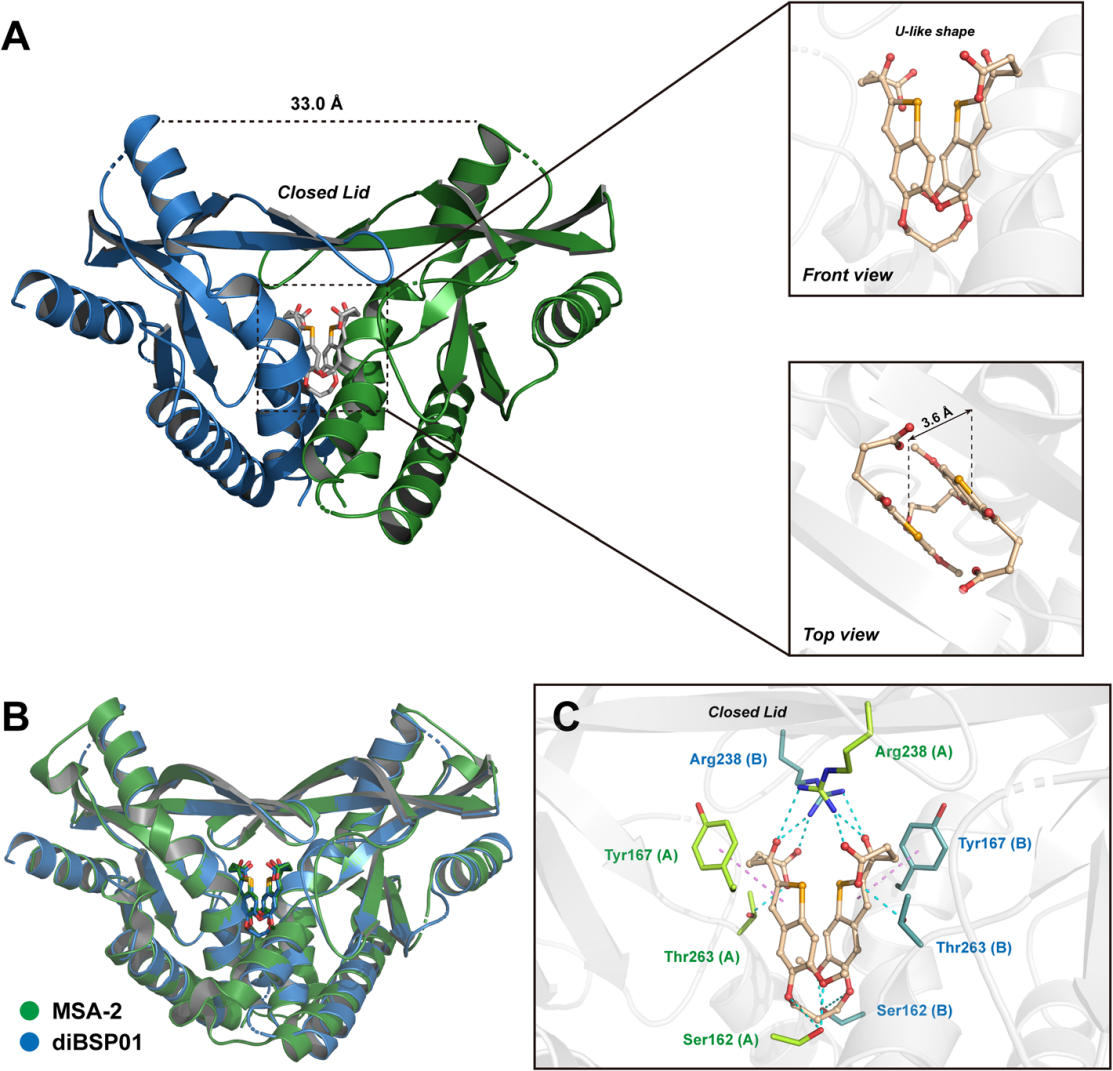
diBSP01 binds to STING as a monomer. (A) The 2.7 Å co-crystal structure of the diBSP01–hSTING^H232^ complex (PDB ID: 7X9P). (B) Illustration of the diBSP01–hSTING^H232^ complex merged with the MSA-2–hSTING^HAQ^ complex (PDB ID: 6UKM). (C) Intricate interactions of diBSP01 with amino acid residues from the STING homodimer (H-bonds colored in cyan and π–π stacking colored in violet).

By merging the diBSP01–STING complex with the MSA-2–STING complex, it is found that diBSP01 coincided with MSA-2 in terms of the spatial location in the pocket and mimicked the interactions in the same way as MSA-2 (Fig. 2B and C). Methoxyl and etheroxyl groups acted as hydrogen bond receptors to form hydrogen bonds with Ser162 located at the bottom of the pocket. In addition, the benzoselenophene rings were parallel to Tyr167, leading to the π–π stacking effect. The folded butyric acid side chains extended into the inside of the protein, where carboxyl and ketone moieties form intricate hydrogen bond networks with Arg238 and Thr263, inducing the formation of a β-sheet lid sealing the pocket and resulting in a ‘closed’ conformation responsible for STING activation and translocation.^4^ In detail, the carbonyl and hydroxyl groups of carboxylic acids interacted with Arg238 of the opposite STING (*e.g.*, chain B) and Thr263 of the proximal STING monomer (*e.g.*, chain A), respectively, while ketones formed the cross-linking hydrogen bonds with Arg238 from the STING homodimer.

Given the promising behaviour of diBSP01 shown in STING's binding properties, the biological evaluation of diBSP01 for activating the STING pathway was subsequently conducted. In the ISRE reporter assay, diBSP01 regained its STING activation efficacy and exhibited similar activity related to MSA-2 with a normalized fold-change value of 34 and 32, respectively (Fig. S5A†). Meanwhile, diBSP01 was effective in cGAS knock-out (KO) ISG-THP1 cells whereas it was incompetent in STING KO ISG-THP1 cells (Fig. S5A†), indicating that diBSP01 reliably targeted the STING adaptor in the cGAS–STING pathway. In THP1 cells, diBSP01 could significantly induce the expression of downstream genes including IL6, CXCL10, and IFNβ and showed a more potent STING activation ability than MSA-2 (Fig. S5B†). Intriguingly, diBSP01 exhibited much more effective gene expression levels at a lower concentration compared with MSA-2 (Fig. S5B†), implying that diBSP01 could still exert its pharmacological potency in a lower dose regimen. Since diBSP01 is a covalent dimer molecule, the equivalent dimer of MSA-2, namely diMSA-2, was prepared (following the same synthetic protocol as for diBSP01) to effectively assess the STING agonism capacity of our proposed compound. As shown in Fig. S5C and D,†diBSP01 exhibited comparable activities to those of diMSA-2 for the activation of the STING pathway in ISG-THP1 cells and the stimulation of downstream gene expression, indicating that diBSP01 was a potent dimer agonist targeting STING.

STING activation by small molecules is a promising strategy for cancer treatment. Nevertheless, systemically administered STING agonists potentially trigger non-negligible on-target immuno-inflammatory responses throughout the whole body as a result of the widespread expression of STING in tumor and normal cells. Indeed, BALB/c mice intraperitoneally administered with 30 mg kg^−1^diBSP01 exhibited complete intolerance, with 90% mortality on the first day and all mice died within 4 days and a reduced dose of 10 mg kg^−1^ also resulted in 90% deaths within 7 days (Fig. S6A†), indicating diBSP01 had a formidable toxicity profile. Consequently, STING activation is urgently needed only in the tumor microenvironment. In view of this, a light-controlled STING agonist modified on the diBSP01 structure was developed as a proof-of-concept.

Given the pivotal role of carboxyl groups in recognizing key amino acid residues (Arg238 and Thr263) in the ligand-binding pocket verified by the co-crystal structure, blocking the terminal carboxyl moieties might be conducive to the temporary abolition of STING agonism. Coumarins are one of the most widely investigated PPGs due to their fast photodeprotection profile under biocompatible conditions and safety considerations upon photorelease.^33^ Therefore, a commonly used diethylaminocoumarinyl-4-methyl (DEACM, Fig. 3) group was selected as the PPG of choice for covalent connection with diBSP01*via* an ester bond. The linking of the two carboxyl groups in diBSP01 to two molecules of DEACM resulted in the first photoactivatable STING agonist caged-diBSP01 (Fig. 3, see the ESI† for the synthesis) that has potential for spatiotemporal control of STING pathway activation by light.

**Fig. 3:**
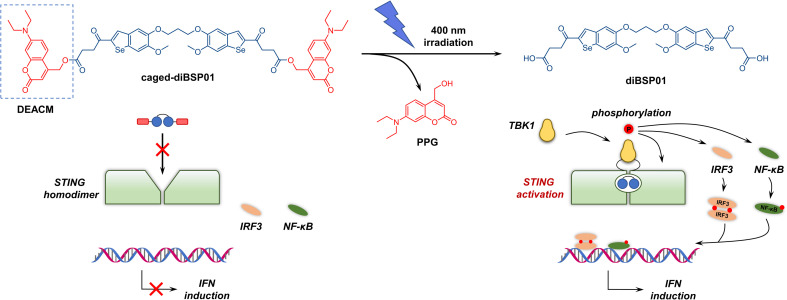
Schematic illustration of the principles of the STING optical control system. Caged-diBSP01 could not bind to and activate STING while 400 nm irradiation led to the liberation of the parent drug diBSP01. Then, diBSP01 could activate the STING-TBK1-IRF3 axis, causing the transcription of downstream IFN genes.

We first investigated the optical properties and photodeprotecting behaviors of caged-diBSP01 (Fig. S7†). In terms of the UV absorption profiles, caged-diBSP01 has two absorption maxima corresponding to the wavelengths of around 340 and 375 nm, which were almost the maximum UV absorption wavelengths of diBSP01 and PPG, respectively (Fig. S7A†). Furthermore, an extremely quick and complete conversion of caged-diBSP01 was observed under 400 nm irradiation using HPLC and UV spectra. The absorption at 375 nm gradually decreased with the extension of irradiation time, suggesting the occurrence of a photouncaging process (Fig. S7B†). The HPLC analysis indicated that caged-diBSP01 could liberate its active form diBSP01 and the corresponding PPG moiety with full conversion within 120 s (Fig. S7C†). It is noteworthy that an intermediate that only deprotected one side of PPGs was observed during this process and it was verified by LC/MS analysis (Fig. S8†), which makes sense mechanistically. Unexpectedly, another by-product with a retention time between those of PPG and diBSP01 was detected, and its molecular weight was determined to be 277 using LC/MS (Fig. S8†). Although we had no idea about the structure of this by-product, we speculated that it was a molecule related to PPG. Meanwhile, caged-diBSP01 was remarkably stable in PBS at 37 °C in the dark over 24 h of monitoring (Fig. S9†). These results together suggested that caged-diBSP01 was capable of causing subsequent STING activation assay under physiological conditions with quite short biocompatible irradiation (400 nm).

To examine whether caged-diBSP01 could activate the STING pathway in a light-controlled manner, the initial study was focused on an ISRE luciferase reporter assay. ISG-THP1 cells were treated with *N*,*N*-dimethylformamide (DMF), PPG, caged-diBSP01, or diBSP01 followed by the absence of or exposure to 400 nm irradiation for 60 s. After 24 h of incubation, a fluorescence signal was detected and normalized to the vehicle control. As shown in Fig. 4A, treatment of caged-diBSP01 with 400 nm light significantly increased the fluorescence intensity compared with that of caged-diBSP01 without irradiation whereas PPG showed no STING activation potency regardless of light exposure. Moreover, caged-diBSP01 treated with 400 nm light for 60 s exhibited the same level of STING pathway activation as diBSP01 did, indicating that caged-diBSP01 was fully converted to its active form diBSP01 in a relatively short duration. In addition, caged-diBSP01 showed time-dependent STING activation properties, which reached its peak within a minute (Fig. 4B).

**Fig. 4:**
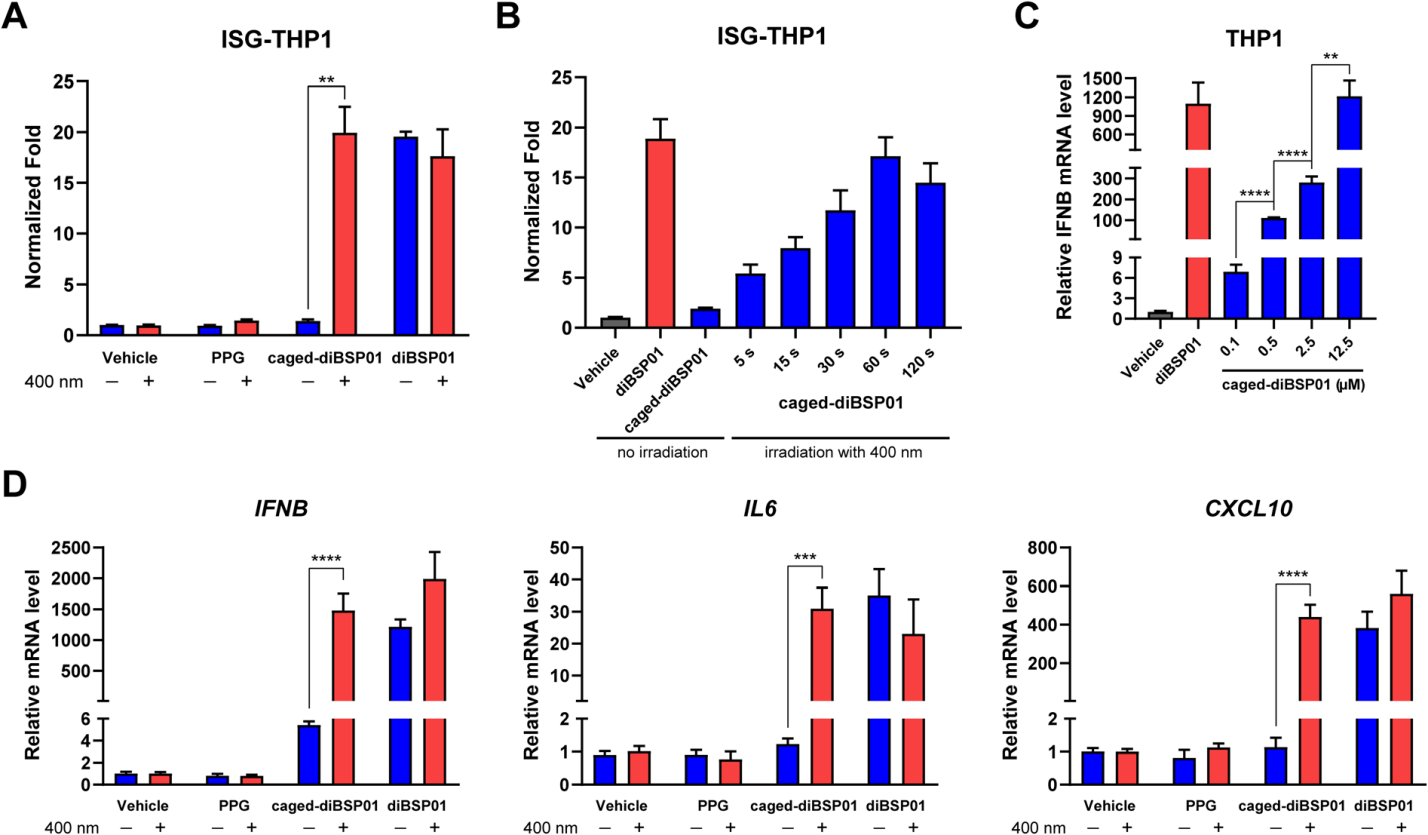
Cell-based activity of caged-diBSP01. (A) ISRE luciferase reporter activity in ISG-THP1 cells treated with the indicated compounds (all 12.5 μM final) in the absence or presence of light (±400 nm, 60 s). The luciferase signal was detected and normalized to the DMF control. (B) ISRE luciferase reporter activity in ISG-THP1 cells incubated with the indicated compounds (all 12.5 μM final) and exposed to 400 nm light for the indicated time periods. (C) Concentration-dependent IFNβ gene expression in THP1 cells treated with DMF, diBSP01 (12.5 μM), and caged-diBSP01 (0.1, 0.5, 2.5, or 12.5 μM) and all groups were irradiated with 400 nm light for 60 s. (D) qRT-PCR analysis of target gene expression in THP1 cells treated with the test article (all 12.5 μM final) in the absence of light or after being irradiated with 400 nm light for 60 s. Data are representative of three independent experiments and values are shown as mean ± SD. Statistical significance was determined by an unpaired Student's *t*-test for (A) and (D), and a one-way analysis of variance (ANOVA) test for (C). **P < 0.01; ***P < 0.001; ****P < 0.0001.

Encouraged by the aforementioned results, the light-controlled expression of downstream genes was determined to further confirm the potency of this photoactivatable agonist. In THP1 cells, the dose-dependent expression of IFNβ was observed after treatment with caged-diBSP01 followed by light stimulation, and the IFNβ mRNA level in the caged-diBSP01 group increased to an identical level compared with that in the diBSP01 group at the same concentration of 12.5 μM (Fig. 4C). This result again demonstrated that caged-diBSP01 could effectively and efficiently liberate its bioactive molecule diBSP01 responsible for the regained STING activation efficacy. Subsequently, the induction of IL6, CXCL10, and IFNβ genes was determined. As expected, the treatment of caged-diBSP01 with 400 nm light significantly induced the expression of all of these three genes while the absence of irradiation showed no activity (Fig. 4D). Similarly, PPG could not activate the STING pathway irrespective of the presence or absence of light (Fig. 4D).

Western blotting experiments were then conducted to visualize the changes of STING downstream proteins so as to verify the protein levels in the cGAS–STING pathway of this photoactivatable system. The results showed that caged-diBSP01 after irradiation remarkably induced the phosphorylation of STING, TBK1, and IRF3. In contrast, caged-diBSP01 without light stimulation could not liberate the active form diBSP01, hence possessing similar levels of protein expression compared with the vehicle control (Fig. 5A). Meanwhile, PPG could still not activate the STING-TBK1-IRF3 axis in the presence or absence of light (Fig. 5A). Furthermore, the regulation of STING and its downstream proteins by caged-diBSP01 treated with light also exhibited dose-dependent properties (Fig. 5B). Together all these assessments indicated that caged-diBSP01 is a promising photo-sensitive STING agonist prodrug which can undergo a rapid and complete release of its parent drug diBSP01 after 400 nm irradiation and was proven to be effective in activating the cGAS–STING pathway in a light-controlled manner.

**Fig. 5:**
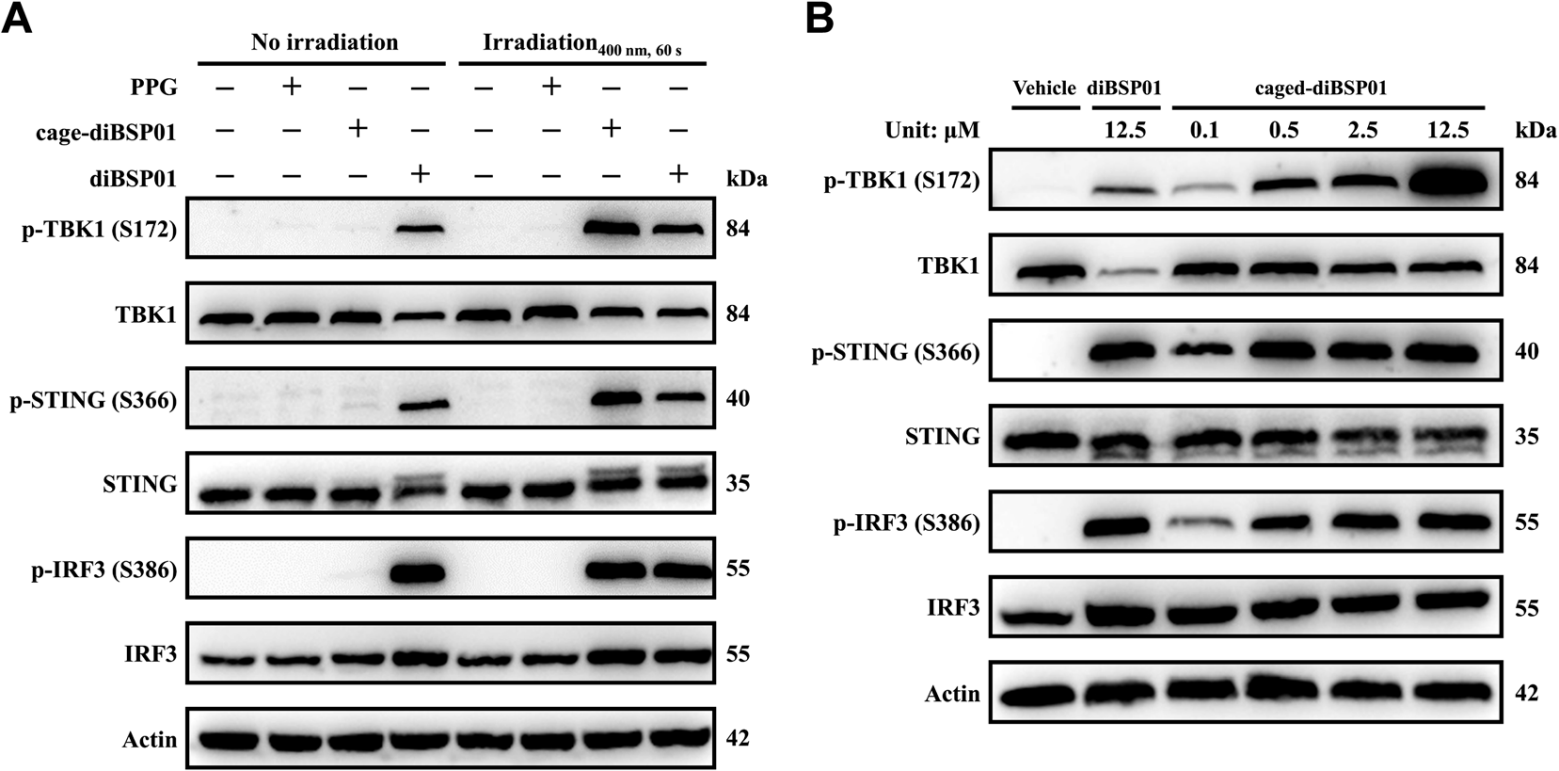
Western blot analysis of the activation of the cGAS–STING pathway controlled by light in THP1 cells. (A) Cells were stimulated with the indicated compounds (all 12.5 μM final) in the absence or presence of light (±400 nm, 60 s). (B) Cells were treated with DMF, diBSP01 (12.5 μM), or caged-diBSP01 (0.1, 0.5, 2.5, or 12.5 μM) and all groups were irradiated with 400 nm light for 60 s. Actin was used as a loading control and data are representative of three replicates.

Zebrafish have been widely used as a model organism for cancer research due to their high fecundity, low cost, accessibility for the modeling of various cancers, and transparent embryos for *in vivo* imaging.^34–36^ They were applied for studying biology related to the STING pathway.^37,38^ Therefore, in this study, we chose zebrafish for developing a tumor model to investigate our photoactivatable STING agonist *in vivo*. Fluorescently labeled MC38 cells were implanted into the yolk sac of zebrafish embryos and zebrafish were incubated with different compounds followed by different light treatments. The fluorescence intensity was detected 4 days after implantation and represented the proliferation of tumor cells. As illustrated in Fig. 6, the caged-diBSP01 group without irradiation showed no significant difference in fluorescence intensity compared with the vehicle group exposed to 400 nm light. The fluorescence intensity significantly decreased after treatment with caged-diBSP01 with 400 nm irradiation for 60 s, suggesting that a light-induced antitumor activity was exerted. Furthermore, MC38-bearing zebrafish were microinjected with compounds into the tumor site and subsequently given the indicated light treatments to better verify our idea of controllable local STING activation. It was found that, compared with the vehicle control, caged-diBSP01 after irradiation could still significantly inhibit tumor growth, but no significant effect was found with caged-diBSP01 in the absence of light (Fig. S10†). Moreover, diBSP01 exhibited almost no antitumor activity with no significant difference compared with the vehicle control in the transiently STING knocked out zebrafish while diBSP01 still showed a significant tumor inhibitory effect in normal wild-type zebrafish (data not shown), which implied that diBSP01 exerted antitumor efficacy in zebrafish in a STING-dependent manner and suggested that diBSP01 could activate the zebrafish STING protein. In conclusion, these results demonstrated that caged-diBSP01 is capable of exerting a light-mediated antitumor potency *in vivo*, offering a new insight into precise STING-dependent innate immune activation and cancer immunotherapy.

**Fig. 6:**
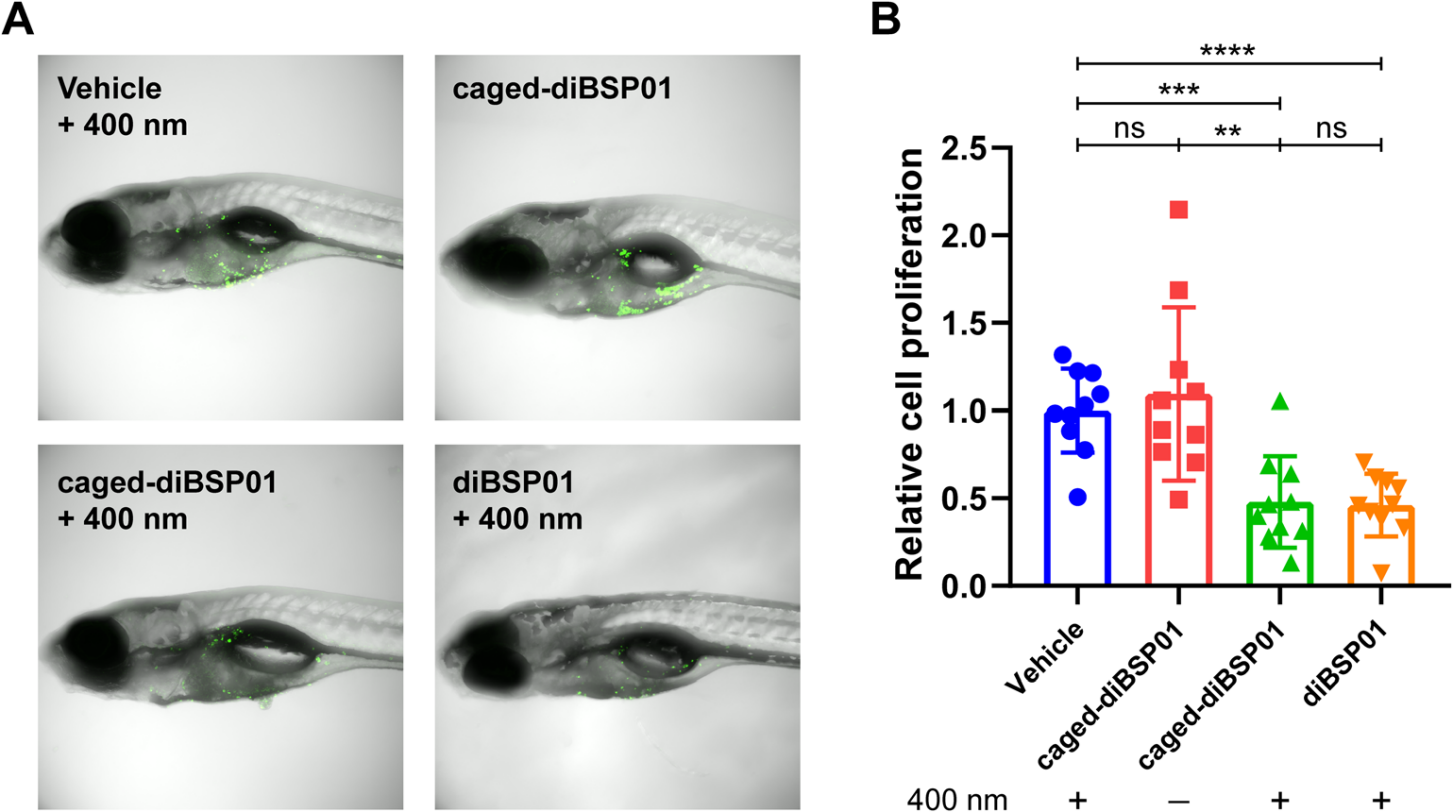
*In vivo* antitumor activity of caged-diBSP01 with systemic administration in a zebrafish xenograft model. (A) Representative images of zebrafish with different treatments as labeled. (B) Zebrafish were incubated with the indicated compounds (all 12.5 μM) followed by different treatments (±400 nm, 60 s). Fluorescence intensity that correlated with the tumor size was detected on 4 days post-implantation and normalized to the vehicle control. Results are shown as mean ± SD (*n* = 10 for each group). Statistical significance was determined by a one-way ANOVA test. ns, not significant; **P < 0.01; ***P < 0.001; ****P < 0.0001.

Meanwhile, BALB/c mice intraperitoneally administered with up to 200 mg kg^−1^ caged-diBSP01 exhibited good tolerance, with no death and the body weight changes consistent with those for the vehicle control (Fig. S6†), indicating that caged-diBSP01 had an improved safety profile after blocking the terminal carboxylic acids of diBSP01. In addition, cell viability studies of dimer diBSP01 and its photoactivatable prodrug caged-diBSP01 were conducted in both MC38 and THP1 cells (Fig. S11†). They showed that both compounds had no cytotoxicity to MC38 and THP1 cells under the tested concentrations within 72 and 24 h of incubation, respectively (Fig. S11A–E†). However, diBSP01 showed significant cytotoxicity to THP1 cells after long-term incubation (72 h). In contrast, caged-diBSP01 caused a lower decrease in cell viability compared with its parent drug diBSP01 (Fig. S11F†), which implied an improvement in the safety of this photo-sensitive prodrug.

In summary, we discovered a novel STING agonist diBSP01 by the covalent dimerization of two monomers of BSP01, a Se-replaced analogue of MSA-2, which resulted in a dramatic change in the STING agonistic potency of these two molecules. BSP01 showed nearly no STING activation ability, while diBSP01 displayed an excellent performance. The affinity assays demonstrated that diBSP01 was a favorable STING binder, which was further illustrated by its co-crystal complex with STING protein. Subsequent structure–activity relationship studies and *in vivo* assessment of this sort of dimerized STING agonist containing the benzo[*b*]selenophene scaffold are ongoing in our laboratory. On the other hand, a photoactivatable STING agonist, caged-diBSP01, was reported for the first time by disguising carboxylic acids with a coumarin-based PPG. Caged-diBSP01 lost its STING activation properties and could efficiently liberate the parent drug diBSP01 which accounted for the restored biological efficacy after light treatment. Furthermore, caged-diBSP01 could exhibit an optically controlled anticancer potency *in vivo* in a zebrafish xenograft model. This concept allows the spatiotemporal activation of STING, which could minimize the adverse effects caused by systemic STING agonism, and offers an option for precise treatment of STING-related diseases such as cancer.

## Data availability

The datasets supporting this article have been uploaded as part of the ESI.†

## Author contributions

Z. Li and J. Bian conceived the project. D. Liu, B. Song, R. Wang, and X. Feng synthesized all compounds and performed the photorelease assessments and analyses. X. Guan and L. Pan performed the co-crystallization experiments. B. Yu, D. Liu, and Z. Wang performed the biological evaluation of compounds. H. Huang, H. Pan, H. Wu, and Z. Qiu performed the toxicity studies in mice. H. Huang, B. Yu and D. Liu conducted the experiments on the zebrafish model. All authors participated in writing, reviewing and editing the manuscript.

## Conflicts of interest

There are no conflicts to declare.

## Supplementary Material

SC-014-D2SC06860E-s001
