# Supplementary material for: Discovery of a photoactivatable dimerized STING agonist based on the benzo[b]selenophene scaffold
Source: Chem Sci. 2023 Mar 13;14(15):4174–82. doi: 10.1039/d2sc06860e (PMC10094158; doi:10.1039/d2sc06860e)
Supplement: SC-014-D2SC06860E-s001 [file SC-014-D2SC06860E-s001.pdf]

Electronic Supplementary Material (ESI) for Chemical Science.
This journal is © The Royal Society of Chemistry 2023

# Supporting Information

## Discovery of a Photoactivatable Dimerized STING Agonist Based on the Benzo[*b*]selenophene Scaffold

*Dongyu Liu,‡[a] Bin Yu,‡[a] Xin Guan,‡[b] Bin Song,[a] Huikai Pan,[a] Renbing Wang,[a] Xi Feng,[a] Lixia Pan,[b] Zhe Wang,[a] Hongxi Wu,[a] Zhixia Qiu,[a] Zhiyu Li,*[a] and Jinlei Bian*[a]*

[a]Jiangsu Key Laboratory of Drug Design and Optimization, Department of Medicinal Chemistry, School of Pharmacy, China Pharmaceutical University, Nanjing 211100, P. R. China

[b]State Key Laboratory of Non-Food Biomass and Enzyme Technology, Guangxi Academy of Sciences, Nanning 530007, P. R. China

‡These authors contributed equally to this work

*Corresponding author: Z. Li (zhiyuli@cpu.edu.cn); J. Bian (bianjl@cpu.edu.cn)

# Table of Contents


Figure S1 Biological evaluation of **BSP01** ....... S3
Figure S2 Dihedral of C2/C3 from **MSA-2** and **BSP01** implies the reason for the decreased activity of **BSP01** ....... S4
Figure S3 Graphical illustration of the strategy for covalent linkage of **BSP01** inspired by spontaneous dimerized **MSA-2** ....... S5
Figure S4 DSF assays of **diBSP01** ....... S6
Figure S5 Cell-based activity of **diBSP01** ....... S7
Figure S6 Toxicity profiles of **diBSP01** and caged-**diBSP01** in mice ....... S8
Figure S7 Optical properties and photorelease behaviors of caged-**diBSP01** ....... S9
Figure S8 LC/MS results of photorelease process after irradiation for 35 s ....... S10
Figure S9 Stability of caged-**diBSP01** in PBS ....... S11
Figure S10 *In vivo* antitumor activity of caged-**diBSP01** with local administration in a zebrafish xenograft model ....... S12
Figure S11 Cell viability of **diBSP01** and caged-**diBSP01** in MC38 and THP1 cells ....... S13
Figure S12 Raw images of Western blot assays ....... S14
Table S1 Data collection and refinement statistics of **diBSP01**–STING complex ....... S15
Chemical protocols ....... S16
- General chemistry ....... S16
- Procedures for the synthesis of **BSP01** ....... S16
- Procedures for the synthesis of **diBSP01** ....... S19
- Procedures for the synthesis of caged-**diBSP01** and PPG ....... S22

Biological protocols ....... S25
- Preparation of recombinant human STING CTD proteins ....... S25
- Differential scanning fluorimetry (DSF) assay ....... S25
- Surface plasmon resonance (SPR) experiment ....... S25
- Isothermal titration calorimetry (ITC) experiment ....... S25
- Crystallization and diffraction data collection ....... S26
- Crystal structure determination ....... S26
- Light-induced photorelease assay ....... S26
- Stability test ....... S27
- Reagents and antibodies ....... S27
- Cell culture ....... S27
- Cell-based luciferase reporter assay ....... S27
- qRT-PCR analysis ....... S28
- Western blotting ....... S28
- Cytotoxicity (CCK-8) assay ....... S29
- Acute toxicity studies in mice ....... S29
- Antitumor studies in zebrafish ....... S30

NMR and HRMS spectra of intermediates and final compounds ....... S31
HPLC spectra of final compounds ....... S44
References ....... S47

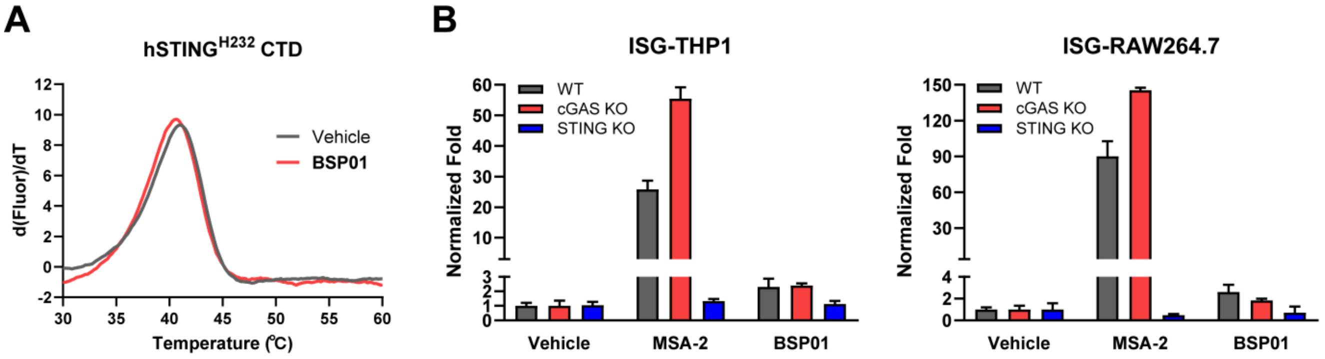


**Figure S1.** Biological evaluation of **BSP01**. (A) **BSP01** showed no binding activity towards hSTING$^{H232}$ at a concentration of 2 mM in differential scanning fluorimetry (DSF) assay. (B) ISRE luciferase reporter activity of **BSP01** (20 μM) in ISG-THP1 cells (left) and ISG-RAW264.7 cells (right). **MSA-2** was selected as a positive control, and fluorescence signal was detected after 24 h of incubation and normalized to DMSO-treated control. Each experiment was conducted three times and values are presented as mean ± standard deviations (SD).

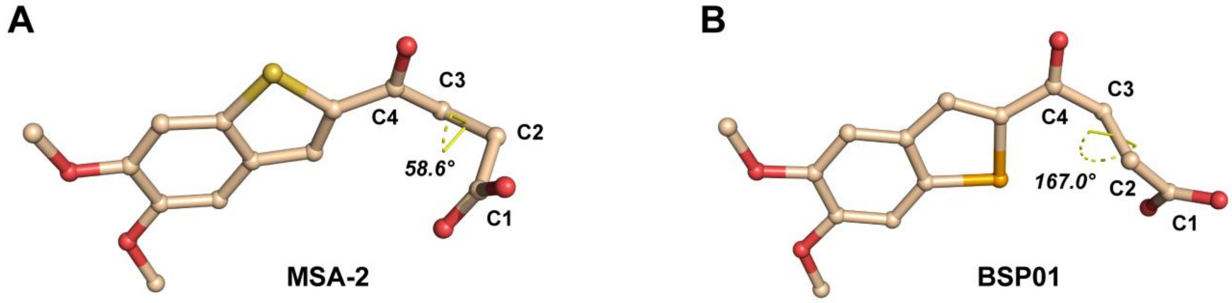


**Figure S2.** Dihedral of C2/C3 from **MSA-2** (PDB ID:6UKM) and **BSP01** is 58.6° (A) and 167.0° (B), respectively. The extremely increased dihedral in **BSP01** is likely to cause an improper folded conformation of butyric acid side chain in the binding pocket and thus losing its activity.

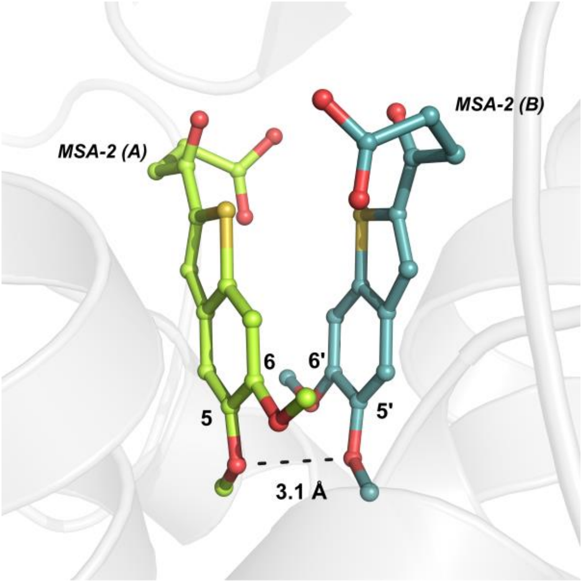


**Figure S3.** The distance of 5-O of one **MSA-2** molecule between 5'-O of the other counterpart is 3.1 Å from the **MSA-2**–STING complex (PDB ID:6UKM), implying a three-carbon atom linker is a good choice that could be used on **BSP01** for covalent dimerization.

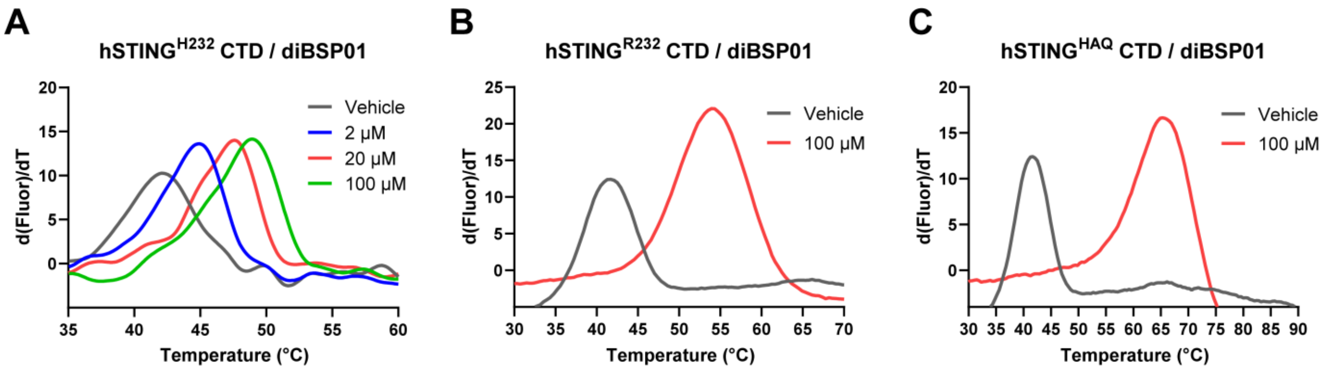


**Figure S4.** DSF assays of **diBSP01**. (A) **diBSP01** could dose-dependently increase the thermostability of hSTING$^{H232}$ CTD protein. **diBSP01** also showed favorable affinity towards hSTING$^{R232}$ (B) and hSTING$^{HAQ}$ (C) protein. Data are representative of three independent experiments.

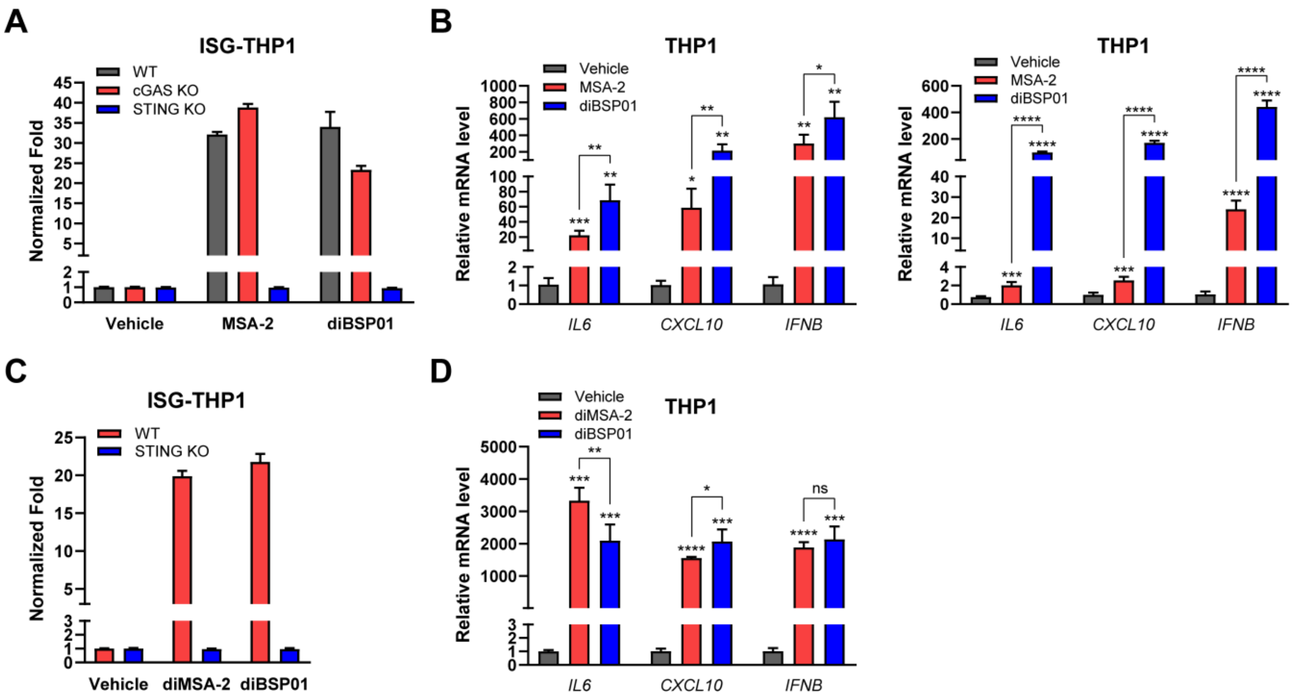


**Figure S5.** Cell-based activity of **diBSP01**. (A) ISG-THP1 cells were treated with DMSO, **MSA-2**, or **diBSP01** (20 μM). Luciferase signal was detected after 24 h of incubation and normalized to DMSO vehicle. (B) qRT-PCR analysis of target gene expression in THP1 cells treated with **MSA-2** or **diBSP01** (25 μM for left and 12.5 μM for right) normalized to DMSO-treated control. (C) ISRE luciferase reporter activity of **diBSP01** compared with **diMSA-2** (20 μM) as described in (A). (D) Target gene expression analysis of **diBSP01** and **diMSA-2** (25 μM) as described in (B). Data are representative of three independent experiments and values indicate mean ± SD. Statistical significance was determined by one-way ANOVA test. ns, not significant; *P < 0.05; **P < 0.01; ***P < 0.001; ****P < 0.0001.

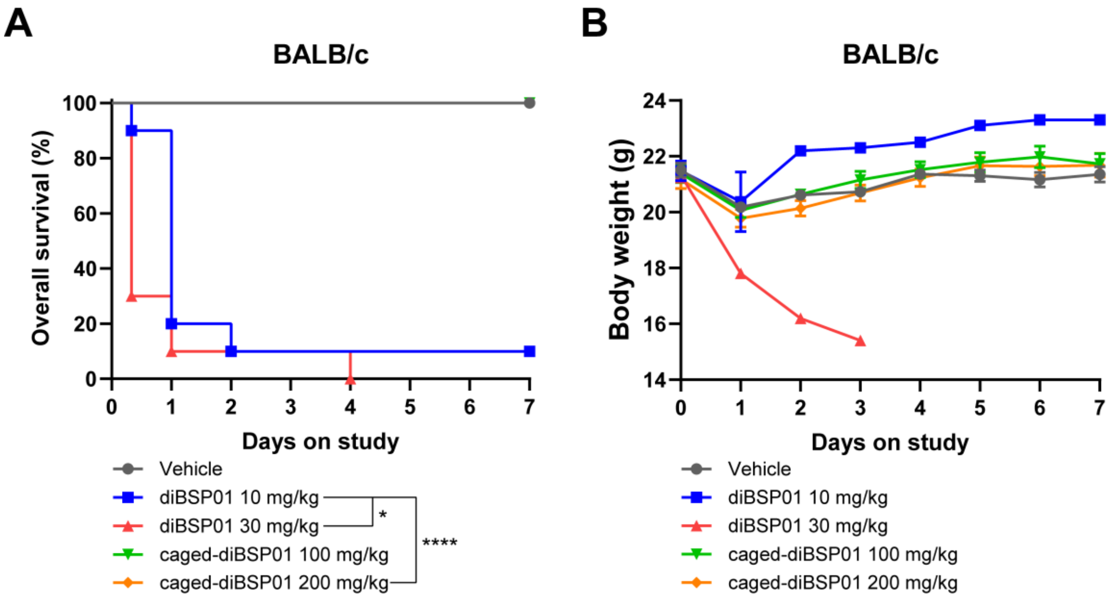


**Figure S6.** Toxicity profiles of **diBSP01** and its photoactivatable prodrug caged-**diBSP01**. BALB/c mice were intraperitoneally administered with indicated compounds at different doses on day 0 followed by monitoring of consecutive 7 days. Toxicity results are presented as survival curves (A) and body weight changes (B). Data indicate mean ± SD. (n = 10 for each group). Statistical significance was determined by log-rank Mantel-Cox test. *$P < 0.05$; ****$P < 0.0001$.

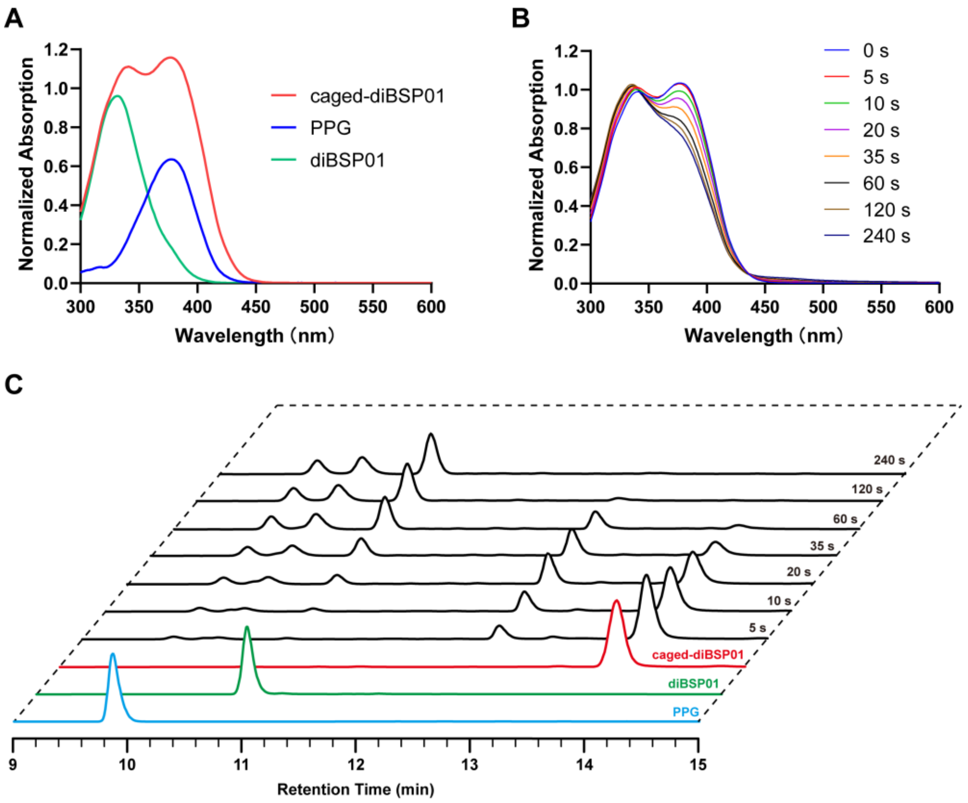


**Figure S7.** Optical properties and photorelease behaviors of caged-**diBSP01**. (A) UV spectra of caged-**diBSP01**, PPG, and **diBSP01**. (B) Absorption changes of caged-**diBSP01** irradiated with 400 nm light for indicated time. (C) HPLC trace of caged-**diBSP01** after time-dependent irradiation with 400 nm light. Experiments were conducted in triplicate.

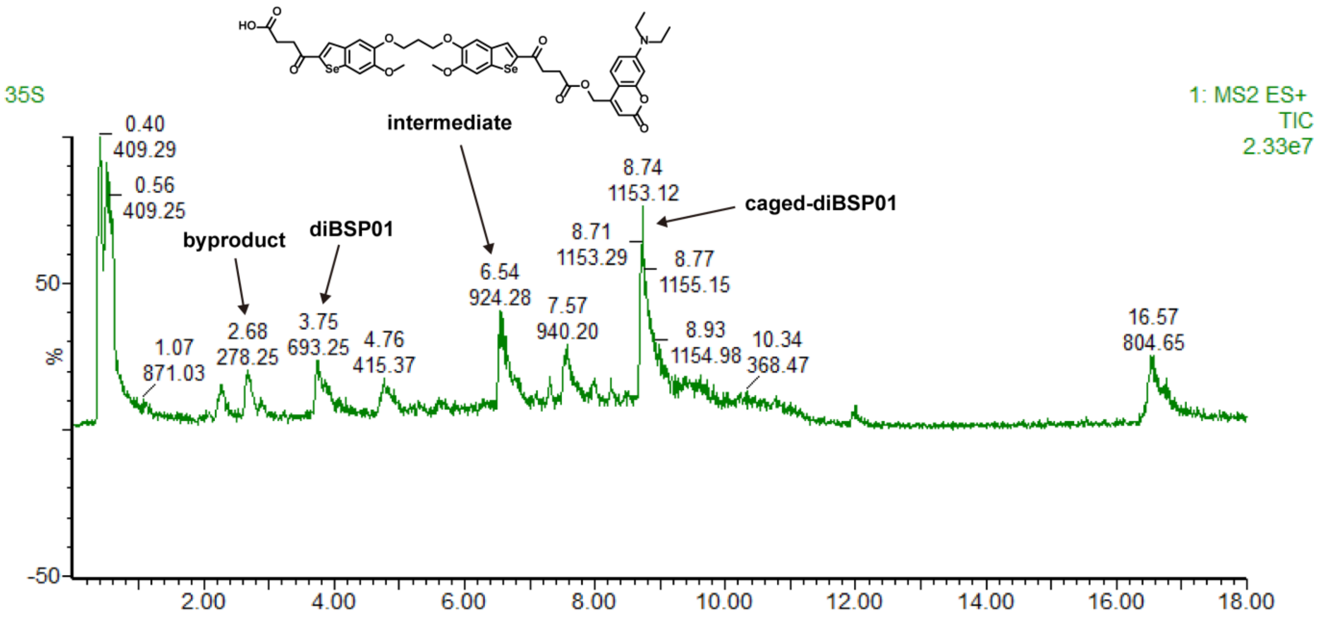


**Figure S8.** LC/MS results of photorelease process after irradiation for 35 s. Under experiment conditions, the procedure generated an intermediate (as shown in figure) that only deprotected one side of PPG and a byproduct that has a molecular weight of 277.

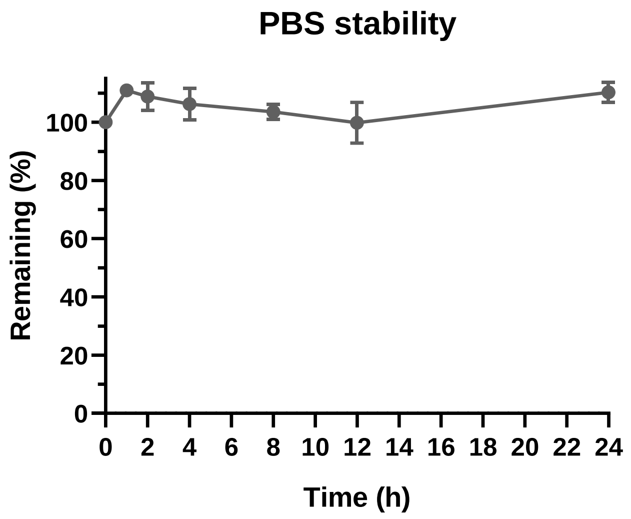


**Figure S9.** Caged-**diBSP01** was prepared in *N*,*N*-dimethylformamide (DMF) as solution (5 mM) and then diluted with PBS buffer (pH 7.4) to a final concentration of 50 μM. Stability test was performed in a dark environment at 37 °C. Experiments were conducted in triplicate and values are expressed as mean ± SD.

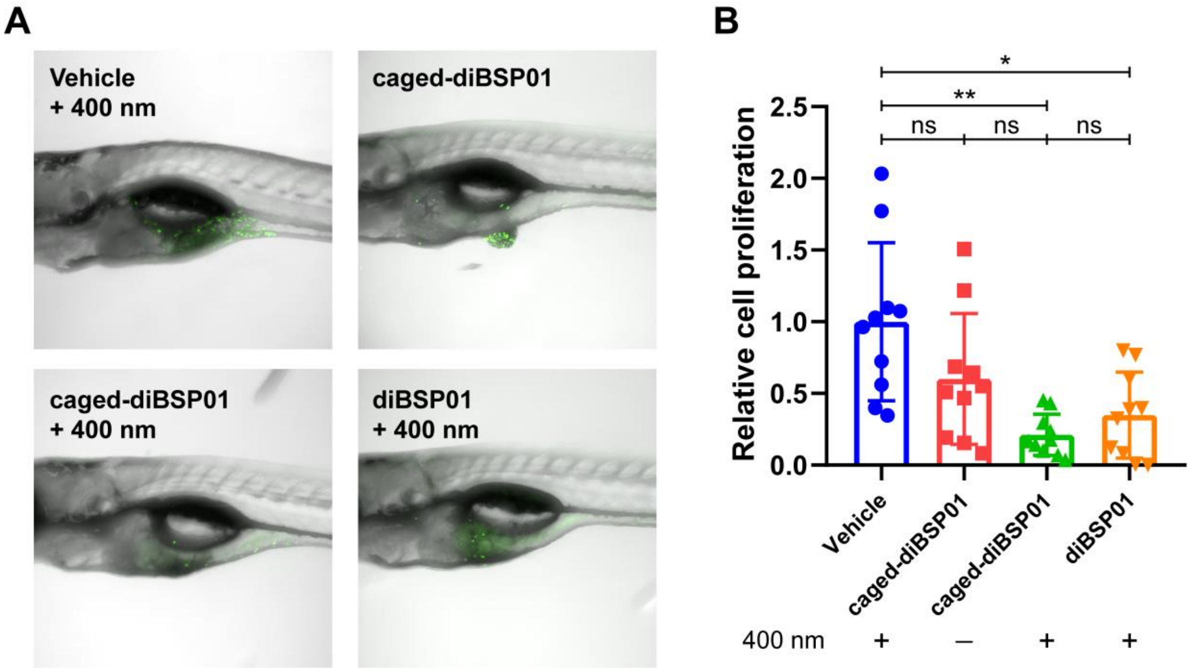


**Figure S10.** *In vivo* antitumor activity of caged-**diBSP01** with local administration in a zebrafish xenograft model. (A) Representative images of zebrafish with different treatments as labeled. (B) Zebrafish were injected with indicated compounds (6.9 ng for **diBSP01** and 11.5 ng for caged-**diBSP01**) followed by different treatments (± 400 nm, 60 s). Fluorescence intensity that corelated to the tumor size was detected on 4 days post-implantation and normalized to vehicle control. Results are shown as mean ± SD (n = 10 for each group). Statistical significance was determined by one-way ANOVA test. ns, not significant; *P < 0.05; **P < 0.01.

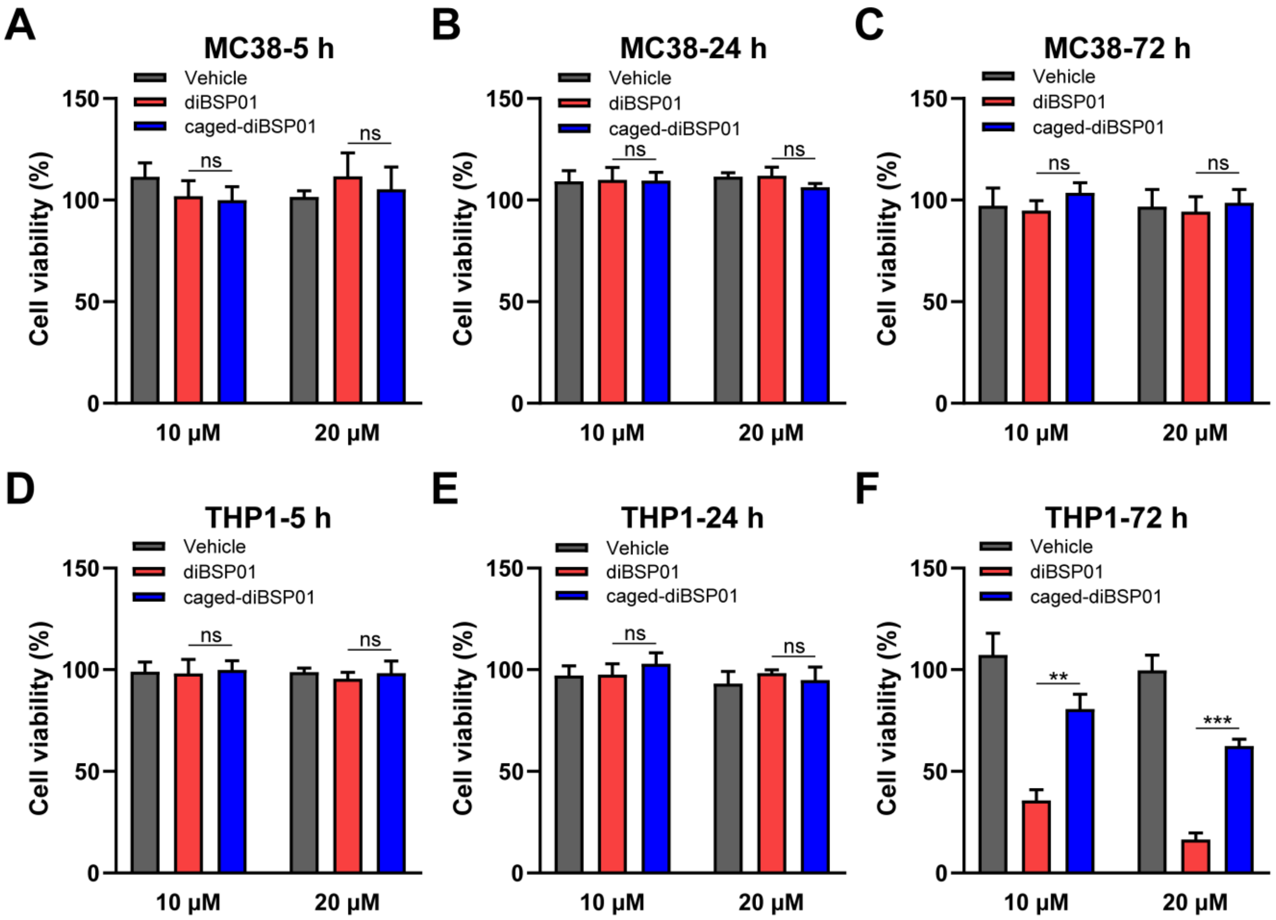


**Figure S11.** Cell viability of **diBSP01** and caged-**diBSP01** in MC38 (A−C) and THP1 (D−F) cells. Cells were cultured and treated with indicated dose of different agents for different time points: 5 h for (A) and (D), 24 h for (B) and (E), and 72 h for (C) and (F). Each experiment was performed three times and results are presented as mean ± SD. Statistical significance was determined by one-way ANOVA test. ns, not significant; $^{**}P < 0.01$; $^{***}P < 0.001$.

**Figure 5A**

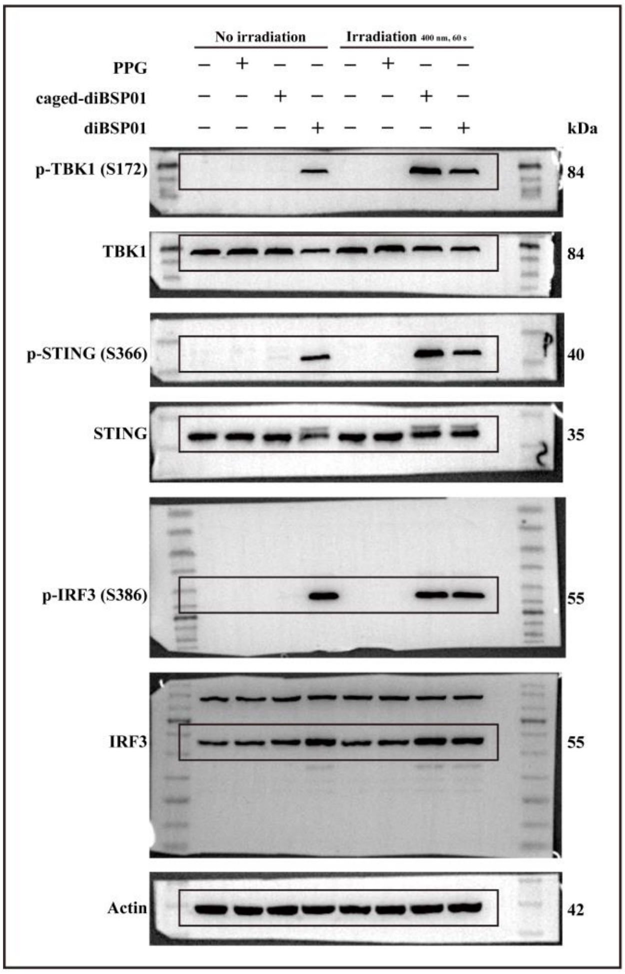


**Figure 5B**

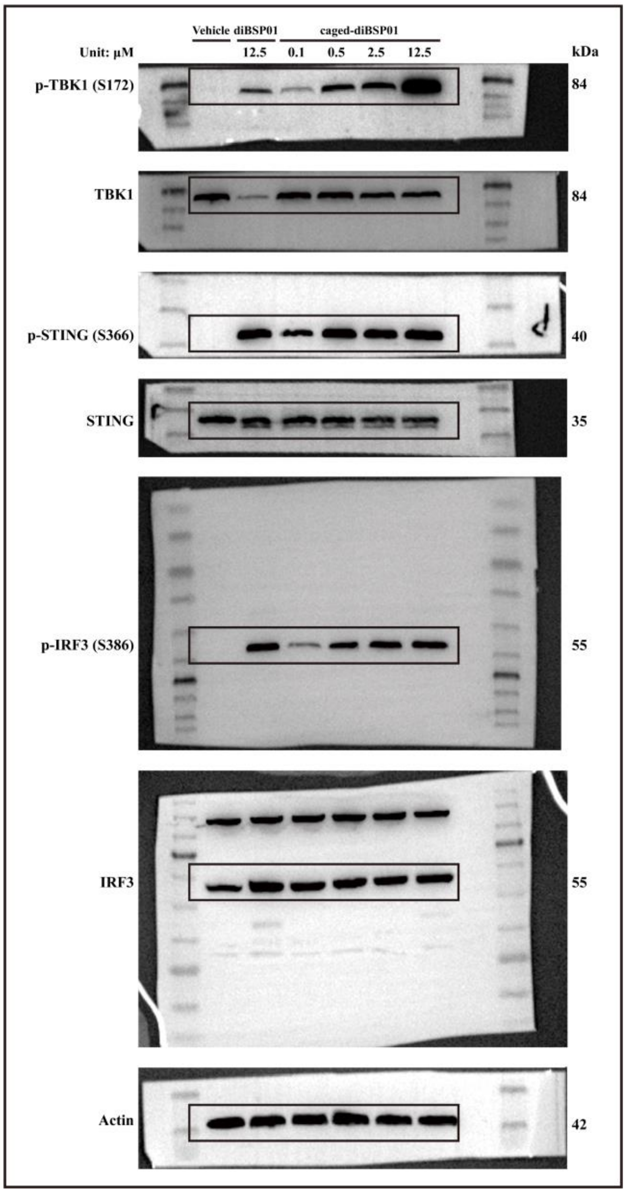


**Figure S12.** Raw images of Western blot assays.

**Table S1.** Data collection and refinement statistics of **diBSP01**−STING complex.

| | **diBSP01**−STING |
|---|---|
| Data collection[a] | |
| Space group | I121 |
| Cell dimensions | |
| *a*, *b*, *c* (Å) | 33.3 54.0 100.0 |
| α, β, γ | 90, 98.5, 90 |
| Resolution (Å)[b] | 49.45-2.70 (2.83-2.70) |
| $R_{merge}$ | 0.101 (0.525) |
| *Rmeas* | 0.142 (0.742) |
| $I / \sigma I$ | 7.0 (3.2) |
| Completeness (%) | 95.1 (96.3) |
| Redundancy | 2.2 (2.3) |
| Refinement | |
| Resolution (Å) | 2.70 |
| No. reflections | 7,915 |
| $R_{work}/R_{free}$ [c] (%) | 24.6/29.5 |
| No. atoms | |
| Protein | 1,331 |
| Ligand/Ion/Water | 41/0/7 |
| *B*-factors (Å$^2$) | |
| Protein | 51.6 |
| Ligand/Ion/Water | 40.8/ /50.6 |
| R.m.s. deviations | |
| Bond lengths (Å) | 0.005 |
| Bond angles (°) | 0.936 |
| Ramachandran stat.[d] | 94.6/5.4/0 |

[a] One crystal was used for data collection.
[b] Values in parentheses are for the highest-resolution shell.
[c] 5% of data are taken for the $R_{free}$ set.
[d] Values are in percentage and are for favored, allowed, and disallowed regions in Ramachandran plots, respectively.

# Chemical protocols

## General chemistry

Unless otherwise noted, all reagents were obtained from commercial suppliers and used without further purification. Reactions were monitored by thin layer chromatography (TLC) visualized under UV light with 254 nm or 365 nm. NMR spectra were measured on a 300 MHz Bruker unit (300 MHz for $^{1}$H NMR, 75 MHz for $^{13}$C NMR) using $CDCl_3$ or DMSO-$d_6$ as the solvent at room temperature. Chemical shifts ($\delta$) are reported in parts per million (ppm) from tetramethylsilane (TMS) using the residual solvent resonance and constants ($J$) are given in hertz. MS spectra were recorded on a LC/MSD TOF HR-MS Spectrum. Column chromatography was performed with 100–200 mesh silica gel and yields refer to chromatographically and spectroscopically pure compounds. All reported compounds are > 95% purity by HPLC analysis.

## Procedures for the synthesis of 4-(5,6-dimethoxybenzo[b]selenophen-2-yl)-4-oxobutanoic acid (BSP01)

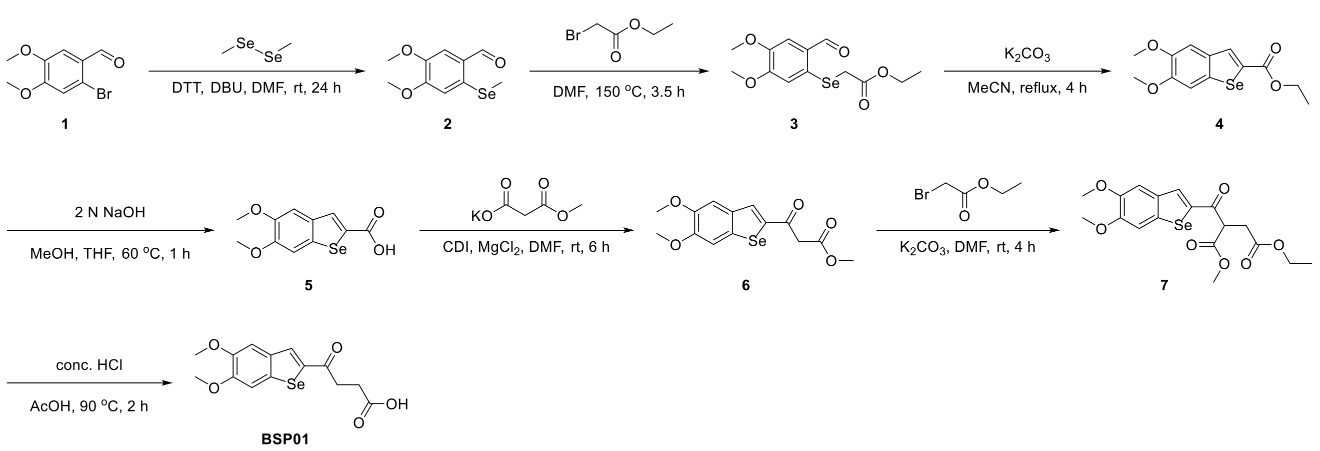


## 4,5-Dimethoxy-2-(methylselanyl)benzaldehyde (2)

Compound **2** was prepared following a published protocol with several adaptations[1]. To a suspension of 2-bromo-4,5-dimethoxybenzaldehyde **1** (11.03 g, 45.0 mmol, 1.5 eq) and DTT (9.24 g, 60.0 mmol, 2.0 eq) in DMF (110 mL) was added dimethyl diselenide (5.64 g, 30.0 mmol, 1.0 eq) and DBU (11.40 g, 75.0 mmol, 2.5 eq). The resulting clear solution was stirred at rt for 24 h under a nitrogen atmosphere. The solution was poured into ice water (500 mL) and the precipitate was collected via filtration, washed with water and purified by silica gel chromatography (petroleum ether : EtOAc = 8 : 1) to obtain the

title compound **2** (6.68 g, 86%) as a pale-yellow solid. $^1$H NMR (300 MHz, $CDCl_3$): $\delta$ = 10.19 (s, 1H), 7.35 (s, 1H), 7.00 (s, 1H), 3.99 (s, 3H), 3.94 (s, 3H), 2.32 (s, 3H) ppm. HRMS (ESI$^+$): cacld for $C_{10}H_{13}O_3Se^+$ $(M + H)^+$ 261.0024; found 261.0023.

**Ethyl 2-((2-formyl-4,5-dimethoxyphenyl)selanyl)acetate (3)**

Compound **3** was prepared according to a modified procedure[2]. To a solution of **2** (5.18 g, 20.0 mmol, 1.0 eq) in DMF (40 mL) was added ethyl bromoacetate (10.02 g, 60.0 mmol, 3.0 eq). The reaction mixture was heated to 150 °C and stirred for 3.5 h. The mixture was cooled down, diluted with water (200 mL) and extracted with EtOAc (50 mL × 3). The organic phase was collected, washed with water (50 mL) and brine (50 mL), dried over anhydrous $Na_2SO_4$, filtered and concentrated *in vacuo* to afford the crude product **3** as a brown liquid, which was used for the next step without further purification. HRMS (ESI$^+$): cacld for $C_{13}H_{17}O_5Se^+$ $(M + H)^+$ 333.0236; found 333.0237.

**Ethyl 5,6-dimethoxybenzo[*b*]selenophene-2-carboxylate (4)**

To a solution of the above crude compound **3** (20.0 mmol, 1.0 eq) in acetonitrile (50 mL) was added $K_2CO_3$ (6.90 g, 50.0 mmol, 2.5 eq). The reaction was heated under reflux with vigorous stirring for 4 h. After cooling down, the mixture was concentrated and the residue was diluted with water (200 mL) and extracted with EtOAc (60 mL × 3). The combined organic layers were washed with water (60 mL) and brine (60 mL), dried over anhydrous $Na_2SO_4$, filtered and concentrated *in vacuo*. The crude product was purified by silica gel chromatography (petroleum ether : EtOAc = 10 : 1) to yield the desired compound **4** (3.57 g, 57% over two steps) as an off-white solid. $^1$H NMR (300 MHz, $CDCl_3$): $\delta$ = 8.18 (s, 1H), 7.32 (s, 1H), 7.28 (s, 1H), 4.40 (q, $J$ = 7.1 Hz, 2H), 3.97 (s, 3H), 3.94 (s, 3H), 1.42 (t, $J$ = 7.1 Hz, 3H) ppm. HRMS (ESI$^+$): calcd for $C_{13}H_{15}O_4Se^+$ $(M + H)^+$ 315.0130; found 315.0125.

**5,6-Dimethoxybenzo[*b*]selenophene-2-carboxylic acid (5)**

To a suspension of **4** (7.21 g, 23.0 mmol) in MeOH (90 mL) and THF (90 mL) was added 2 N NaOH aqueous solution (35 mL). The reaction mixture was stirred at 60 °C for 1 h, during which the reaction gradually became clear. Upon cooling to rt, the solvent was removed under reduced pressure and the residue was adjusted to pH = 2~3 using 1 N HCl. The precipitate was collected via filtration, washed with water and dried *in vacuo* at 30 °C overnight to get the desired product **5** (6.50 g, 99%) as an off-

white solid. $^1$H NMR (300 MHz, $CDCl_3$): $\delta$ = 8.28 (s, 1H), 7.34 (s, 1H), 7.31 (s, 1H), 3.98 (s, 3H), 3.95 (s, 3H) ppm. HRMS (ESI$^-$): calcd for $C_{11}H_9O_4Se^-$ $(M - H)^-$, 284.9672; found 284.9675.

**Methyl 3-(5,6-dimethoxybenzo[*b*]selenophen-2-yl)-3-oxopropanoate (6)**

To a solution of **5** (7.13 g, 25.0 mmol, 1.0 eq) in DMF (140 mL) was added CDI (12.15 g, 75.0 mmol, 3.0 eq). The mixture was stirred at rt for 1 h followed by addition of $MgCl_2$ (7.13 g, 75.0 mmol, 3.0 eq) and monomethyl monopotassium malonate (11.70 g, 75.0 mmol, 3.0 eq). After stirring at rt for an additional 5 h, the reaction mixture was poured into water (400 mL). The precipitate was filtered, washed with water and purified by silica gel chromatography (petroleum ether : EtOAc = 8 : 1) to afford the title compound **8** (7.18 g, 84%) as a yellow solid. $^1$H NMR (300 MHz, $CDCl_3$): $\delta$ = 8.10 (s, 1H), 7.33 (s, 1H), 7.30 (s, 1H), 4.00 (s, 2H), 3.98 (s, 3H), 3.95 (m, 3H), 3.77 (s, 3H) ppm. HRMS (ESI$^+$): calcd for $C_{14}H_{15}O_5Se^+$ $(M + H)^+$, 343.0079; found 343.0076.

**4-Ethyl 1-methyl 2-(5,6-dimethoxybenzo[*b*]selenophene-2-carbonyl)succinate (7)**

To a mixture of **6** (150 mg, 0.44 mmol, 1.0 eq) and $K_2CO_3$ (91 mg, 0.66 mmol, 1.5 eq) in DMF (4 mL) was added ethyl bromoacetate (88 mg, 0.53 mmol, 1.2 eq). The reaction was vigorously stirred at rt for 4 h prior to being diluted with water (50 mL). The mixture was extracted with EtOAc (12 mL × 3) and the organic phase was collected, washed with water (12 mL) and brine (12 mL), dried over anhydrous $Na_2SO_4$, filtered and concentrated *in vacuo* to get the desired compound **9** as crude, which was best used without further purification. HRMS (ESI$^+$): calcd for $C_{18}H_{21}O_7Se^+$ $(M + H)^+$, 429.0447; found 429.0445.

**4-(5,6-Dimethoxybenzo[*b*]selenophen-2-yl)-4-oxobutanoic acid (BSP01)**

To a solution of the above crude compound **7** (0.44 mmol) in AcOH (2 mL) was slowly added conc. HCl (2 mL). After stirring at 90 °C for 2 h, the reaction mixture was cooled down, diluted with water (60 mL) and then extracted with EtOAc (15 mL × 3). The combined organic layers were washed with water (15 mL) and brine (15 mL), dried over anhydrous $Na_2SO_4$, filtered and concentrated *in vacuo*. The residue was purified by silica gel chromatography (DCM : MeOH = 30 : 1) to give the desired compound **BSP01** (100 mg, 67% over two steps) as a pale-yellow solid. $^1$H NMR (300 MHz, DMSO-*d*$_6$): $\delta$ = 8.43 (s, 1H), 7.70 (s, 1H), 7.53 (s, 1H), 3.85 (s, 3H), 3.82 (s, 3H), 3.27–3.23 (m, 2H), 2.60–2.56 (m, 2H) ppm. $^{13}$C NMR (75 MHz, DMSO-*d*$_6$): $\delta$ = 193.99, 174.14, 150.77, 148.81, 145.06, 137.15,

135.46, 134.61, 109.36, 108.37, 56.24, 56.01, 32.98, 28.49 ppm. HRMS (ESI$^-$): cacld for $C_{14}H_{13}O_5Se^-$ $(M - H)^-$, 340.9934; found 340.9935.

**Procedures for the synthesis of 4,4'-((propane-1,3-diylbis(oxy))bis(6-methoxybenzo[b]selenophene-5,2-diyl))bis(4-oxobutanoic acid) (diBSP01)**

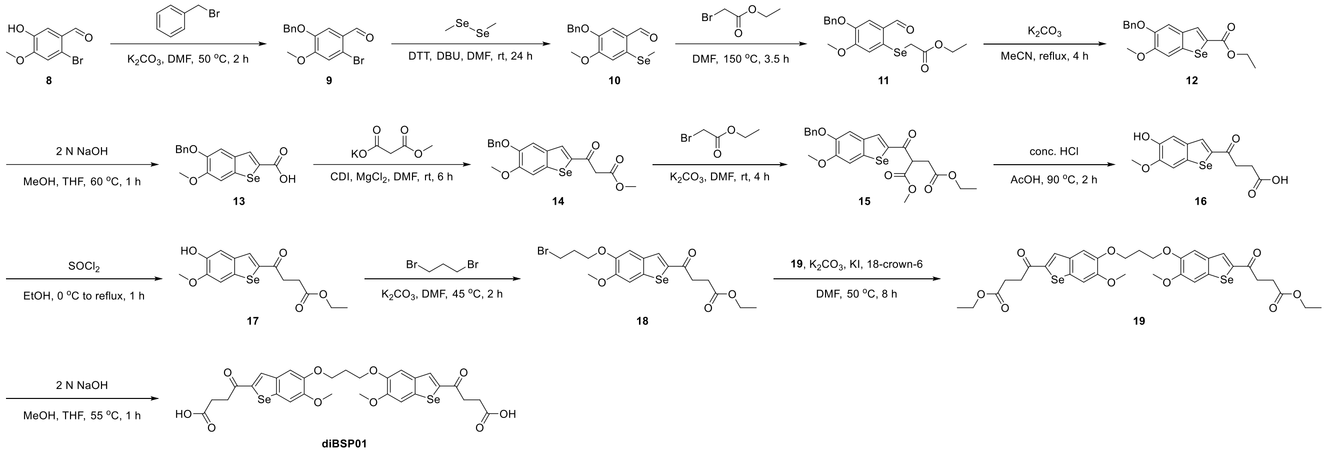


**5-(Benzyloxy)-2-bromo-4-methoxybenzaldehyde (9)**

To a mixture of 2-bromo-5-hydroxy-4-methoxybenzaldehyde **8** (6.93 g, 30.0 mmol, 1.0 eq) and $K_2CO_3$ (8.28 g, 60.0 mmol, 2.0 eq) in DMF (300 mL) was added benzyl bromide (6.12 g, 36.0 mmol, 1.2 eq). The reaction was stirred at 50 °C for 2 h before being poured into ice water (1.5 L). The precipitate was filtered, washed with water and then dried *in vacuo* at 30 °C overnight. The crude material was suspended in petroleum ether (200 mL) and stirred at rt for 20 min. The insoluble substance was collected via filtration and wash with petroleum ether to give the title compound **9** (9.24 g, 96%) as a white solid. $^1$H NMR (300 MHz, $CDCl_3$): $\delta$ = 10.16 (s, 1H), 7.48 (s, 1H), 7.46–7.30 (m, 5H), 7.07 (s, 1H), 5.16 (s, 2H), 3.95 (s, 3H) ppm. HRMS (ESI$^+$): cacld for $C_{15}H_{14}BrO_3^+$ $(M + H)^+$, 321.0121; found 321.0122.

**5-(Benzyloxy)-4-methoxy-2-(methylselanyl)benzaldehyde (10)**

Compound **10** was prepared according to the protocol of compound **2**. Yield = 87%, pale-yellow solid. $^1$H NMR (300 MHz, $CDCl_3$): $\delta$ = 10.08 (s, 1H), 7.46–7.30 (m, 6H), 6.99 (s, 1H), 5.18 (s, 2H), 3.99 (s, 3H), 2.31 (s, 3H) ppm. HRMS (ESI$^+$): cacld for $C_{16}H_{17}O_3Se^+$ $(M + H)^+$, 337.0337; found 337.0339.

**Ethyl 2-((4-(benzyloxy)-2-formyl-5-methoxyphenyl)selanyl)acetate (11)**

Compound **11** was prepared according to the protocol of compound **3**. **11** was used as crude for the subsequent step. HRMS (ESI$^+$): cacld for $C_{19}H_{21}O_5Se^+$ (M + H)$^+$, 409.0549; found 409.0552.

**Ethyl 5-(benzyloxy)-6-methoxybenzo[*b*]selenophene-2-carboxylate (12)**

Compound **12** was prepared according to the protocol of compound **4**. Yield = 76% over two steps, pale-yellow solid. $^1$H NMR (300 MHz, DMSO-$d_6$): $\delta$ = 8.24 (s, 1H), 7.74 (s, 1H), 7.68 (s, 1H), 7.50–7.32 (m, 5H), 5.12 (s, 2H), 4.33 (q, $J$ = 6.9 Hz, 2H), 3.86 (s, 3H), 1.33 (t, $J$ = 7.0 Hz, 3H) ppm. HRMS (ESI$^+$): cacld for $C_{19}H_{19}O_4Se^+$ (M + H)$^+$, 391.0443; found 391.0447.

**5-(Benzyloxy)-6-methoxybenzo[*b*]selenophene-2-carboxylic acid (13)**

Compound **13** was prepared according to the protocol of compound **5**. Yield = 99%, off-white solid. $^1$H NMR (300 MHz, DMSO-$d_6$): $\delta$ = 8.16 (s, 1H), 7.73 (s, 1H), 7.66 (s, 1H), 7.50–7.32 (m, 5H), 5.12 (s, 2H), 3.85 (s, 3H) ppm. HRMS (ESI$^-$): cacld for $C_{17}H_{13}O_4Se^-$ (M – H)$^-$, 360.9985; found 360.9988.

**Methyl 3-(5-(benzyloxy)-6-methoxybenzo[b]selenophen-2-yl)-3-oxopropanoate (14)**

Compound **14** was prepared according to the protocol of compound **6**. Yield = 84%, yellow solid. $^1$H NMR (300 MHz, $CDCl_3$): $\delta$ = 8.02 (s, 1H), 7.48–7.31 (m, 7H), 5.21 (s, 2H), 3.98 (s, 3H), 3.97 (s, 2H), 3.76 (s, 3H) ppm. HRMS (ESI$^+$): cacld for $C_{20}H_{19}O_5Se^+$ (M + H)$^+$, 419.0392; found 419.0389.

**4-Ethyl 1-methyl 2-(5-(benzyloxy)-6-methoxybenzo[*b*]selenophene-2-carbonyl)succinate (15)**

Compound **15** was prepared according to the protocol of compound **7**. **15** was used as crude for the next step without further purification. HRMS (ESI$^+$): cacld for $C_{24}H_{25}O_7Se^+$ (M + H)$^+$, 505.0760; found 505.0761.

**4-(5-Hydroxy-6-methoxybenzo[*b*]selenophen-2-yl)-4-oxobutanoic acid (16)**

Compound **16** was prepared according to the protocol of compound **BSP01**. Yield = 59% over two steps, pale-yellow solid. $^1$H NMR (300 MHz, DMSO-$d_6$): $\delta$ = 12.16 (s, 1H), 9.36 (s, 1H), 8.39 (s, 1H), 7.63 (s, 1H), 7.36 (s, 1H), 3.86 (s, 3H), 3.26 (t, $J$ = 6.1 Hz, 2H), 2.60 (t, $J$ = 6.3 Hz, 2H) ppm. HRMS (ESI$^-$): cacld for $C_{13}H_{11}O_5Se^-$ (M – H)$^-$, 326.9777; found 326.9775.

**Ethyl 4-(5-hydroxy-6-methoxybenzo[*b*]selenophen-2-yl)-4-oxobutanoate (17)**

To a stirred suspension of **16** (500 mg, 1.53 mmol, 1.0 eq) in anhydrous ethanol (15 mL) at 0 °C was slowly added $SOCl_2$ (546 mg, 4.59 mmol, 3.0 eq). The resulting reaction mixture was allowed to warm to rt spontaneously and then heat to reflux. After an additional 1 h of stirring, the reaction was cooled down and the solvent was removed under reduced pressure. The residue was diluted with water (60 mL) and extracted with EtOAc (15 mL × 3). The combined organic phase was washed with water (15 mL) and brine (15 mL), dried over anhydrous $Na_2SO_4$, filtered and concentrated *in vacuo*. The crude product was then purified by by silica gel chromatography (petroleum ether : EtOAc = 4 : 1) to afford the desired compound **17** (516 mg, 95%) as an off-white solid. $^1$H NMR (300 MHz, $CDCl_3$): $\delta$ = 8.08 (s, 1H), 7.39 (s, 1H), 7.31 (s, 1H), 4.20 (q, $J$ = 7.1 Hz, 2H), 3.99 (s, 3H), 3.35 (t, $J$ = 6.7 Hz, 2H), 2.79 (t, $J$ = 6.8 Hz, 2H), 1.29 (t, $J$ = 7.1 Hz, 3H) ppm. HRMS (ESI$^+$): cacld for $C_{15}H_{17}O_5Se^+$ $(M + H)^+$, 357.0236; found 357.0234.

**Ethyl 4-(5-(3-bromopropoxy)-6-methoxybenzo[*b*]selenophen-2-yl)-4-oxobutanoate (18)**

To a mixture of **17** (258 mg, 0.73 mmol, 1.0 eq) and $K_2CO_3$ (201 mg, 1.45 mmol, 2.0 eq) in DMF (7 mL) was added 1,3-dibromopropane (1.47 g, 7.27 mmol, 10.0 eq). The reaction was stirred at 45 °C for 2 h before being diluted with water (50 mL) and extracted with EtOAc (12 mL × 3). The combined organic layers were washed with water (12 mL) and brine (12 mL), dried over anhydrous $Na_2SO_4$, filtered and concentrated *in vacuo*. The residue was purified by silica gel chromatography (petroleum ether : EtOAc = 4 : 1) to yield the title compound **18** (263 mg, 76%) as a pale-yellow solid. $^1$H NMR (300MHz, $CDCl_3$): $\delta$ = 8.11 (s, 1H), 7.34 (s, 1H), 7.33 (s, 1H), 4.23–4.13 (m, 4H), 3.95 (s, 3H), 3.69 (t, $J$ = 6.3 Hz, 2H), 3.35 (t, $J$ = 6.8 Hz, 2H), 2.80 (t, $J$ = 6.7 Hz, 2H), 2.46–2.38 (m, 2H), 1.29 (t, $J$ = 7.1 Hz, 3H) ppm. HRMS (ESI$^+$): cacld for $C_{18}H_{22}BrO_5Se^+$ $(M + H)^+$, 476.9810; found 476.9808.

**Diethyl 4,4'-((propane-1,3-diylbis(oxy))bis(6-methoxybenzo[*b*]selenophene-5,2-diyl))bis(4-oxobutanoate) (19)**

To a mixture of **18** (250 mg, 0.53 mmol, 1.0 eq), **17** (186 mg, 0.53 mmol, 1.0 eq) and $K_2CO_3$ (217 mg, 1.58 mmol, 3.0 eq) in DMF (6 mL) was added KI (9 mg, 0.05 mmol, 0.1 eq) and 18-crown-6 (14 mg, 0.05 mmol, 0.1 eq). The reaction mixture was stirred at 50 °C for 8 h followed by being diluted with water (50 mL) and extracted with EtOAc (12 mL × 3). The organic phase was collected, washed with water (12 mL) and brine (12 mL), dried over anhydrous $Na_2SO_4$, filtered and concentrated *in vacuo*. The crude material was purified by silica gel chromatography (petroleum ether : EtOAc = 2 : 1) to give

the title compound **19** (347 mg, 88%) as a tawny solid. $^{1}$H NMR (300 MHz, DMSO-$d_6$): $\delta$ = 8.40 (s, 2H), 7.72 (s, 2H), 7.59 (s, 2H), 4.23 (t, $J$ = 5.6 Hz, 4H), 4.09 (q, $J$ = 7.1 Hz, 4H), 3.85 (s, 6H), 3.31 (t, $J$ = 5.8 Hz, 4H), 2.68 (t, $J$ = 6.2 Hz, 4H), 2.33–2.27 (m, 2H), 1.20 (t, $J$ = 7.1 Hz, 6H) ppm. HRMS (ESI$^+$): cacld for $C_{33}H_{37}O_{10}Se_2^+$ (M + H)$^+$, 753.0712; found 753.0712.

**4,4'-((Propane-1,3-diylbis(oxy))bis(6-methoxybenzo[*b*]selenophene-5,2-diyl))bis(4-oxobutanoic acid) (diBSP01)**

To a suspension of **19** (150 mg, 0.20 mmol) in MeOH (2 mL) and THF (2 mL) was added 2 N NaOH aqueous solution (0.6 mL). The reaction was heated to 55 °C and stirred for 1 h. The mixture was cooled to rt and the solvent was removed under reduced pressure followed by being adjusted to pH = 2~3 with 1 N HCl. The resulting mixture was extracted with EtOAc (15 mL × 3) and the combined organic phase was washed with water (15 mL) and brine (15 mL), dried over anhydrous $Na_2SO_4$, filtered and concentrated *in vacuo*. The residue was purified by silica gel chromatography (DCM : MeOH = 30 : 1) to give the desired product **diBSP01** (98 mg, 71%) as a yellow solid. $^{1}$H NMR (300 MHz, DMSO-$d_6$): $\delta$ = 12.19 (s, 2H), 8.40 (s, 2H), 7.71 (s, 2H), 7.59 (s, 2H), 4.23 (t, $J$ = 5.9 Hz, 4H), 3.85 (s, 6H), 3.27 (t, $J$ = 5.9 Hz, 4H), 2.61 (t, $J$ = 6.2 Hz, 4H), 2.31–2.27 (m, 2H) ppm. $^{13}$C NMR (75 MHz, DMSO-$d_6$): δ = 194.04, 174.20, 150.99, 147.94, 145.12, 137.39, 135.52, 134.61, 110.74, 108.60, 65.63, 56.34, 33.01, 29.08, 28.50 ppm. HRMS (ESI$^+$): cacld for $C_{29}H_{29}O_{10}Se_2^+$ (M + H)$^+$, 697.0086; found 697.0090.

**Procedures for the synthesis of bis((7-(diethylamino)-2-oxo-2H-chromen-4-yl)methyl) 4,4'-((propane-1,3-diylbis(oxy))bis(6-methoxybenzo[b]selenophene-5,2-diyl))bis(4-oxobutanoate) (caged-diBSP01) and 7-(diethylamino)-4-(hydroxymethyl)-2H-chromen-2-one (PPG)**

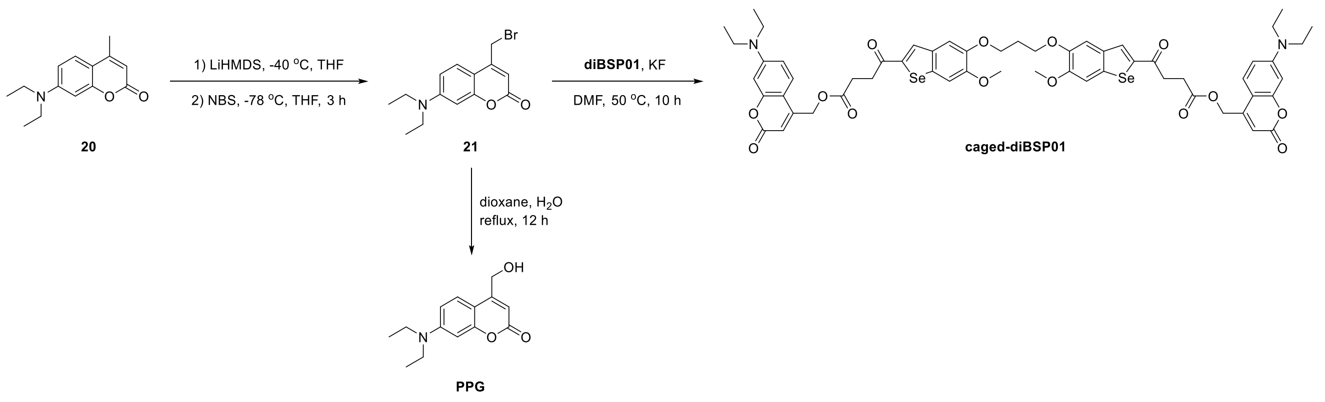


**4-(Bromomethyl)-7-(diethylamino)-2*H*-chromen-2-one (21)**

Compound **21** was prepared according to a modified protocol[3]. To a flame-dried three-necked flask equipped with magnetic stir bar was added 7-(diethylamino)-4-methyl-2H-chromen-2-one **20** (1.85 g, 8.0 mmol, 1.0 eq) and anhydrous THF (80 mL). After cooling to –40 °C, 1 M LiHMDS in THF (20 mL, 2.5 eq) was added via syringe over 20 min under an atmosphere of nitrogen followed by cooling to −78 °C. NBS (1.57 g, 8.8 mmol, 1.1 eq) dissolved in 20 mL anhydrous THF was added to the flask via syringe over 30 min. The reaction mixture was allowed to stirred at −78 °C for 3 h and then quenched with saturated $NH_4Cl$ aqueous solution (120 mL). The mixture was concentrated *in vacuo*, diluted with water (100 mL) and extracted with EtOAc (50 mL × 3). The combined organic phase was washed with water (50 mL) and brine (50 mL), dried over anhydrous $Na_2SO_4$, filtered and concentrated *in vacuo*. The crude product was purified by silica gel chromatography (petroleum ether : EtOAc = 6 : 1) to give the desired compound **21** (1.84 g, 74%) as an orange solid. $^1H$ NMR (300 MHz, $CDCl_3$): $\delta$ = 7.51 (d, $J$ = 9.0 Hz, 1H), 6.65 (dd, $J_1$ = 9.0 Hz, $J_2$ = 2.4 Hz, 1H), 6.52 (d, $J$ = 2.4 Hz, 1H), 6.14 (s, 1H), 4.40 (s, 2H), 3.46 (q, $J$ = 7.1 Hz, 4H), 1.24 (t, $J$ = 7.1 Hz, 6H) ppm. HRMS ($ESI^+$): cacld for $C_{14}H_{17}BrNO_2^+$ $(M + H)^+$, 310.0437; found 310.0440.

**bis((7-(diethylamino)-2-oxo-2H-chromen-4-yl)methyl) 4,4'-((propane-1,3-diylbis(oxy))bis(6-methoxybenzo[b]selenophene-5,2-diyl))bis(4-oxobutanoate) (caged-diBSP01)**

To a mixture of **21** (201 mg, 0.65 mmol, 3.0 eq) and KF (75 mg, 1.30 mmol, 6.0 eq) in DMF (5 mL) was added **diBSP01** (150 mg, 0.22 mmol, 1.0 eq). The resulting reaction mixture was stirred at 50 °C for 10 h. The mixture was diluted with water (50 mL) and extracted with DCM (20 mL × 3). The combined organic phase was washed with water (20 mL) and brine (20 mL), dried over anhydrous $Na_2SO_4$, filtered and concentrated *in vacuo*. The crude product was purified by silica gel chromatography (DCM : MeOH = 100 : 1) to yield the title compound (63 mg, 25%) as a pale-yellow solid. $^1H$ NMR (300 MHz, $CDCl_3$): $\delta$ = 8.04 (s, 2H), 7.35 (s, 2H), 7.32 (s, 2H), 7.31 (d, $J$ = 9.2 Hz, 4H), 6.60 (d, $J$ = 8.9 Hz, 2H), 6.53 (s, 2H), 6.15 (s, 2H), 5.25 (s, 4H), 4.34 (t, $J$ = 5.8 Hz, 4H), 3.93 (s, 6H), 3.43–3.33 (m, 12H), 2.92 (t, $J$ = 6.5 Hz, 4H), 2.49–2.41 (m, 2H), 1.22 (t, $J$ = 7.0 Hz, 12H) ppm. $^{13}C$ NMR (75 MHz, $CDCl_3$): $\delta$ = 192.59, 172.09, 161.87, 156.25, 151.15, 150.54, 149.33, 148.00, 144.95, 138.17, 135.06, 133.05, 124.58, 110.28, 108.89, 107.33, 106.81, 106.24, 97.97, 65.77, 61.80, 56.19, 44.88, 32.89, 29.11, 28.37, 12.41 ppm. HRMS ($ESI^+$): cacld for $C_{57}H_{59}N_2O_{14}Se_2^+$ $(M + H)^+$, 1155.2291; found 1155.2294.

**7-(diethylamino)-4-(hydroxymethyl)-2H-chromen-2-one (PPG)**

PPG was prepared following a reported procedure[3]. To a solution of **21** (0.50 g, 1.60 mmol) in dioxane (8 mL) was added deionized water (8 mL). The reaction was stirred and heated under reflux for 12 h. The reaction was cooled down, diluted with water (60 mL) and extracted with EtOAc (15 mL × 3). The organic layers were collected, washed with water (15 mL) and brine (15 mL), dried over anhydrous $Na_2SO_4$, filtered and concentrated *in vacuo*. The residue was purified by silica gel chromatography (petroleum ether : EtOAc = 2 : 1) to afford the title product (0.25 g, 63%) as an orange solid. $^1$H NMR (300 MHz, $CDCl_3$): $\delta$ = 7.33 (d, $J$ = 8.9 Hz, 1H), 6.58 (d, $J$ = 8.8 Hz, 1H), 6.50 (s, 1H), 6.28 (s, 1H), 4.84 (s, 2H), 3.44 (q, $J$ = 7.0 Hz, 4H), 1.22 (t, $J$ = 7.1 Hz, 6H) ppm. $^{13}$C NMR (75 MHz, $CDCl_3$): $\delta$ = 163.03, 156.01, 155.40, 150.44, 124.39, 108.64, 106.31, 105.15, 97.60, 60.76, 44.70, 12.40 ppm. HRMS (ESI$^+$): cacld for $C_{14}H_{18}NO_3^+$ $(M + H)^+$, 248.1281; found 248.1282.

## Biological protocols

### Preparation of recombinant human STING CTD proteins

Recombinant $hSTING^{H232}$ CTD, $hSTING^{R232}$ CTD, and $hSTING^{HAQ}$ CTD proteins were expressed in *E. coli* BL21 (DE3) codon plus RIL strain and strains were stimulated with 0.1 mM IPTG at 16 °C for 18–20 h. Purification of proteins was performed on Ni-NTA resin (Cytiva) and then size-exclusion chromatography (Cytiva). Protein was concentrated to ~10 mg/ml by using AmiconUltra-15 centrifugal filters (10,000 MWCO). The purified protein was analyzed by sodium dodecyl sulfate polyacrylamide gel electrophoresis (SDS-PAGE) under denatured conditions.

### Differential scanning fluorimetry (DSF) assay

In general, the hSTING CTD protein was diluted with Tris buffer (20 mM Tris pH 7.4, 150 mM NaCl) to obtain a final concentration of 5 μM and 1:1000 (v/v) SYPRO Orange dye was added to the protein solution. To 96-well optical plate was added 19 μL of the above solution in triplicate followed by 1 μL of the test article of indicated concentration or vehicle control. The plate was incubated at 37 °C for 30 min before being subjected to a real time PCR cycler (QuantStudio 3, ThermoFisher). The fluorescence intensity was recorded and the first derivative of thermal denaturation curves was used to calculate to $T_m$ of STING protein.

### Surface plasmon resonance (SPR) experiment

SPR experiments were conducted using a Biacore T200 system (GE Healthcare) at 25 °C. The $hSTING^{H232}$ CTD protein was diluted to 10 μg/mL in 10 mM sodium acetate (pH 5.5) and captured on a Series S Sensor Chip CM5 (at a flow rate of 10 μL/min and a contact time of 420 s) to a final capture level of ~9000 resonance units (RU). Compound **diBSP01** was prepared in PBS-P buffer (GE Healthcare) containing 5% DMSO. A series of sample dilutions (3333–13.7 nM, 6 steps, 3-fold) were sequentially injected in to the system at a flow rate of 30 μL/min and contact and dissociation times of 90 s. The binding sensorgrams were all calibrated by solvent correction (5% DMSO) and the binding affinity values were calculated using Biacore T200 Evaluation Software 2.0 according to the manufacturer's instruction.

### Isothermal titration calorimetry (ITC) experiment

The isothermal titration calorimetry was performed using a MicroCal$^{TM}$ ITC200 (GE Healthcare Life Sciences, USA) calorimeter. The titrations of hSTING$^{H232}$ CTD protein (30 μM in 20 mM Tris pH 7.5, 150 mM NaCl and 5% DMSO) and **diBSP01** (500 μM) were conducted at 25 °C with a syringe stirring speed of 1000 rpm and 20 successive injections with a pre-injection delay of 120 s. The data were post-processed by Origin 2018 software, and the enthalpy change ($\Delta H$), entropy change ($\Delta S$), and affinity constant $K_a$ ($K_a = 1/K_D$) were calculated.

**Crystallization and diffraction data collection**

Purified hSTING$^{H232}$ with the His-Tag at 0.346 mM concentration was mixed with ligand **diBSP01** at 75 mM concentration (1:216.76 ratio), and then subjected to crystallization. Co-crystallization with ligand **diBSP01**, resulted in crystals of different size and morphology grown by vapor-diffusion sitting drop method from crystallization condition (0.2 M KCl, 20% (w/v) PEG3350). All diffraction data were collected at 100–110 K at the Shanghai Synchrotron Radiation Facility (SSRF) BL 17U1 beam line. Glycerol mixed the reservoir solution was used as a cryoprotectant at concentration 20%. The images were integrated, and intensities merged by using Aquarium[4].

**Crystal structure determination**

Molecular replacement was used to determine all crystal structures. All reflections were used for refinement. The final atomic models were obtained after a number of iterations of refinement using phenix.refine[5] and manual model building with the program Coot[6]. Diffraction data, refinement statistics, and quality of the structure are summarized in Table S1.

**Light-induced photorelease assay**

All compounds were dissolved in DMF as solution (5 mM) and stored at -20 °C. Test compound was diluted in a mixed solution (DMF : MeOH : PBS= 2 : 2 : 1, v/v/v) at a final concentration of 50 μM and then irradiated by a light. In all subsequent photolysis experiments, illumination of the test articles was performed at a 400 nm light source at a light intensity of 6.5 mW/cm$^2$ for the indicated time. Samples were taken at indicated time and directly subjected to HPLC analysis or UV spectra detection. Absorption spectra of samples were investigated by MOLECULAR DEVICES SpectraMax i3. The scanning wavelength ranged from 300 to 600 nm with a step size of 1 nm, and the absorbance of

compounds was normalized to vehicle control. The standard absorption curves of PPG, **diBSP01**, and caged-**diBSP01** were measured under the same solvent conditions and concentrations.

**Stability test**

Compound caged-**diBSP01** was diluted with PBS (pH 7.4) buffer to obtain a final concentration of 50 μM and incubated at 37 °C in the dark. Samples were taken at indicated time, diluted with a triple volume of solvent (DMF : MeOH = 1 : 1, v/v) and then subjected to HPLC analysis.

**Reagents and antibodies**

Compounds were dissolved in dimethyl formamide (DMF, aladdin) at a concentration of 5 mM or dissolved in dimethyl sulfoxide (DMSO, aladdin) at a concentration of 10 mM and then diluted with the RPMI 1640 medium (KeyGEN, Nanjing, China) containing 10% fetal bovine serum (FBS, VivaCell). Normocin, Zeocin, QUANTI-Luc luciferase reagent were purchased from InvivoGen. Primary antibodies against TBK1 (#38066S), phosphor-TBK1 (Ser172, #5483T), IRF3 (#4302S), phosphor-IRF3 (Ser396, #29047S), STING (#13647S), and phosphor-STING (Ser366, #50907T) were purchased from Cell Signaling Technology (CST, USA). β-ACTIN, the anti-mouse IgG horseradish peroxidase (HRP)-linked secondary antibody and the anti-rabbit IgG horseradish peroxidase (HRP)-linked secondary antibody were purchased from FDbio (Fudebio, Hangzhou, China).

**Cell culture**

The THP1-Lucia ISG cells and THP1-Dual KO-STING cells were purchased from InvivoGen and maintained in growth media consisting of RPMI 1640, 2mM L-glutamine, 25 mM HEPES, 10% heat-inactivated FBS, 100 U/mL penicillin, 50 μg/mL streptomycin, and 100 μg/mL Normocin. To maintain luciferase expression, 100 μg/mL of Zeocin was added to the growth medium every other. THP1 cells were purchased from American Type Culture Collection (ATCC, USA) and maintained in growth media consisting of RPMI 1640, 2mM L-glutamine, 25 mM HEPES, 10% heat-inactivated FBS, 100 U/mL penicillin, 50 μg/mL streptomycin, and 0.05 mM 2-Mercaptoethanol. All of the cells were maintained at 37 °C with 5% $CO_2$ atmosphere.

**Cell-based luciferase reporter assay**

THP1-Lucia ISG cells ($5 \times 10^5$ cells/mL) that were seeded into each well of 48-well plates and incubated with an indicated concentration range of the test article were irradiated with 400 nm light for indicated time and incubated at 37 °C for 24 hours under a dark condition. DMSO or DMF was added as the negative control. To evaluate expression of the luciferase reporter, 50 μL of cells were added into a 96-well white opaque plates and 50 μl of Quanti-Luc luciferase reagent was added to each well and luminescence was read using an Envision plate reader set with an integration time of 0.1 seconds. For each cell type, luminescence signals for test article samples were normalized to vehicle-treated samples and reported as relative light units (RLU).

**qRT-PCR analysis**

The cellular activation of STING signaling pathway leads to *IFNβ*, *IL6*, and *CXCL10* mRNA expression in THP1 cells and was quantified using qRT-PCR. Total RNAs was isolated from $1.5 \times 10^6$ THP1 cells per well of a 12-well plate, which were incubated with test article (**MSA-2**, **diBSP01**, PPG, or caged-**diBSP01**) or vehicle (DMSO or DMF) with or without indicated irradiation (400 nm) and harvested with 3 h. RNA was extracted using Trizol reagent (Vazyme Biotech Co., ltd, Nanjing, China) and quantified using a Nano-Drop 2000 spectrophotometer. Reverse transcription was carried out with a HiScript II Q RT SuperMix for qPCR (+gDNA wiper) Kit (Vazyme Biotech Co., ltd, Nanjing, China) according to the manufacturer's instructions. Subsequently, the product from reverse transcription was amplified with a Taq pro Universal SYBR qPCR Master Mix Kit (Vazyme Biotech Co., ltd, Nanjing, China) using Applied Biosystems. Primer sequences were as follows: *IFN-β*_F: 5'-TGGCTGGAATGAGACTATTGTT-3', *IFN-β*_R: 5'-GGTAATGCAGAATCCTCCCATA-3'; *IL-6*_F: 5'- CACTGGTCTTTTGGAGTTTGAG-3', *IL-6*_R: 5'-GGACTTTTGTACTCATCTGCAC-3'; *CXCL-10*_F: 5'-CTCTCTCTAGAACTGTACGCTG-3', *CXCL-10*_R: 5'-ATTCAGACATCTCTTCTCACCC-3', *β-Actin*_F: 5'-CAGATGTGGATCAGCAAGCAGGAG-3', *β-Actin*_R: 5'-CGCAACTAAGTCATAGTCCGCCTAG-3'.

All reactions were performed in triplicate, and analysis of the relative gene expression level was normalized to β-Actin using the $2^{-\Delta\Delta Ct}$ method. Melting curves were routinely performed to determine the specificity of the PCR.

**Western blotting**

THP1 cells were cultured in 6-well plates and treated with various treatment groups (**diBSP01**, PPG, or caged-**diBSP01**) in designed concentrations with or without indicated irradiation (400 nm). Then the Petri dishes were incubated at 37 °C with 5 % CO2 for 3 h. After 3 h, Cells were solubilized in 1X protein lysis buffer (50 mM Tris, pH 7.4, 150 mM NaCl, 1% Triton X-100, 1% sodium deoxycholate, 0.1% SDS) with freshly added protease and phosphatase inhibitors (RIPA : PMSF = 99 : 1) (Fudebio, Hangzhou, China) and uniformed by the Pierce BCA protein assay kit (Fudebio, Hangzhou, China). Equal protein amounts were loaded to 10% SDS-PAGE and transferred to a nitrocellulose membrane (Merk millipore, USA), which was blocked with 5% non-fat milk (Fudebio, Hangzhou, China) and then incubated overnight at 4 °C with primary antibodies: anti-TBK1 (dilution, 1:1000), anti-p-TBK1 (Ser172) (dilution, 1:1000), anti-IRF3 (dilution, 1:1000), anti-p-IRF3 (Ser396) (dilution, 1:1000), anti-STING (dilution, 1:1000), anti-p-STING (Ser366) (dilution, 1:1000), and anti-β-Actin (dilution, 1:4000). Subsequently, the bands were incubated with HRP-conjugated anti-rabbit IgG (dilution, 1:5000) or HRP-conjugated anti-mouse IgG (dilution, 1:5000) at room temperature for 2 h. Chemistar High-sig ECL Western Blotting Substrate Kit (Tanon, Shanghai, China) was used to visualize the protein bands and the protein levels were quantified by the gray values of the bands in the resulting images using ImageJ.

**Cytotoxicity (CCK-8) assay**

Compound **diBSP01** and caged-**diBSP01** were assessed for their cytotoxicity against two type cell lines, including THP1 and MC38, using the cell counting kit-8 (CCK-8) assay. Briefly, cells were seeded into 96-well plates and incubated with an indicated concentration of the test articles at 37 °C for different time. After treatment with test articles, CCK8 was added and the cells were incubated for another 3 h. Cell viability was detected with a microplate reader at a wavelength of 450 nm.

**Acute toxicity studies in mice**

The experimental procedures, and animal use and care protocols were performed according to the guidelines approved by the Animal Welfare and Ethics Committee (AWEC) of China Pharmaceutical University. BALB/c mice were randomly divided into five groups (for each group, n = 10, half male and half female) and each group was intraperitoneally administered with vehicle, **diBSP01** (10 mg/kg or 30 mg/kg), or caged-**diBSP01** (100 mg/kg or 200 mg/kg). Compounds were prepared in a formulation of 5% DMSO, 40% PEG 400, and 55% PBS pH 7.4. After single administration, mice were monitored

and weighed daily for consecutive 7 days. After 7 days, all mice were given euthanasia. Toxicity results are presented as survival curves and body weight changes and the statistical differences in survival curves were determined by log-rank Mantel-Cox test using GraphPad software 8.0.

**Antitumor studies in zebrafish**

The facility for zebrafish is accredited by the Association for Assessment and Accreditation of Laboratory Animal Care (AAALAC) international. Dio (Vybrant™) labeled MC38 cells were implanted into the yolk sac of Tg(coro1a:DsRed) transgenic zebrafish of 2 days post-fertilization at 100 cells per fish. Zebrafish were incubated at 34 °C in an illumination incubator (14 h of light and 10 h of dark for a cycle) for 24 h and then randomly divided into four groups. Each group was incubated with DMF, **diBSP01**, or caged-**diBSP01** (12.5 μM) or injected with DMF, **diBSP01**, or caged-**diBSP01** (6.9 ng for **diBSP01** and 11.5 ng for caged-**diBSP01**) into the yolk sac, followed by light irradiation (400 nm, 60 s) or avoiding light. All groups were then incubated at 34 °C in a dark condition for another 72 h. Stereo fluorescence microscope (OLYMPUS MVX10) and confocal microscope (Nikon AX) were used to take pictures of zebrafish and the fluorescence intensity of MC38 cells was analyzed with the ImageJ software.

# NMR and HRMS spectra of intermediates and final compounds

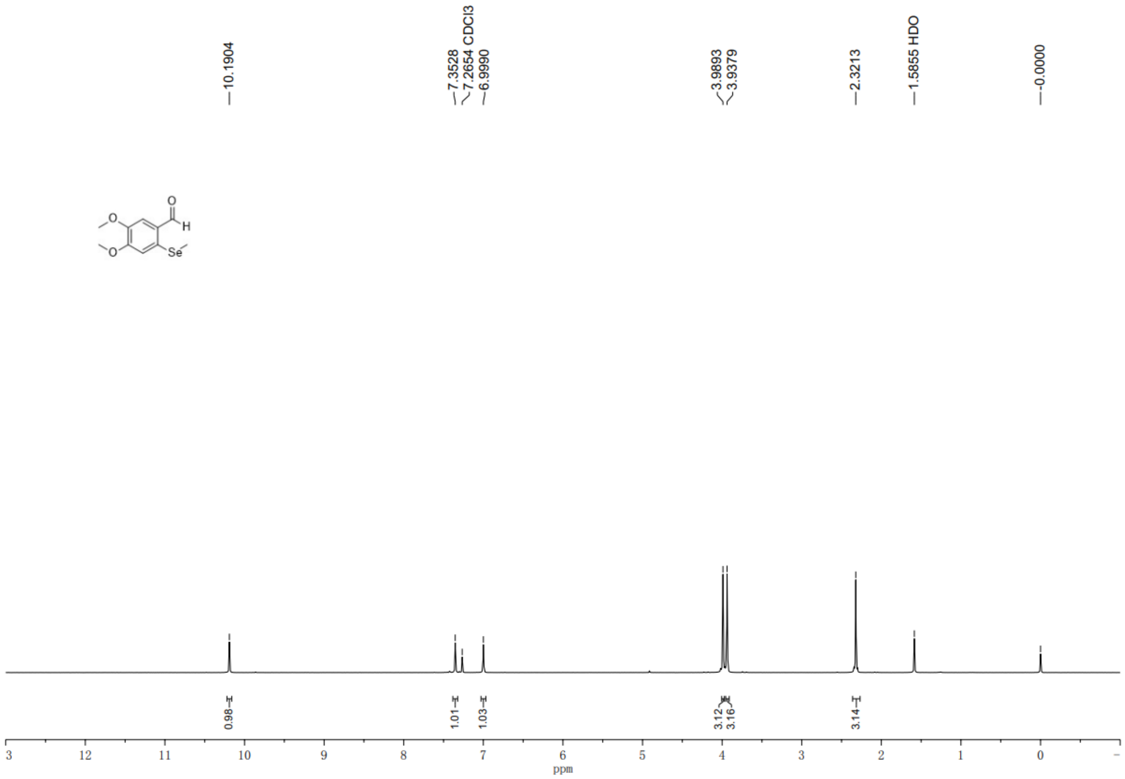

$^{1}$H NMR of compound **2** (300 MHz, $CDCl_3$)

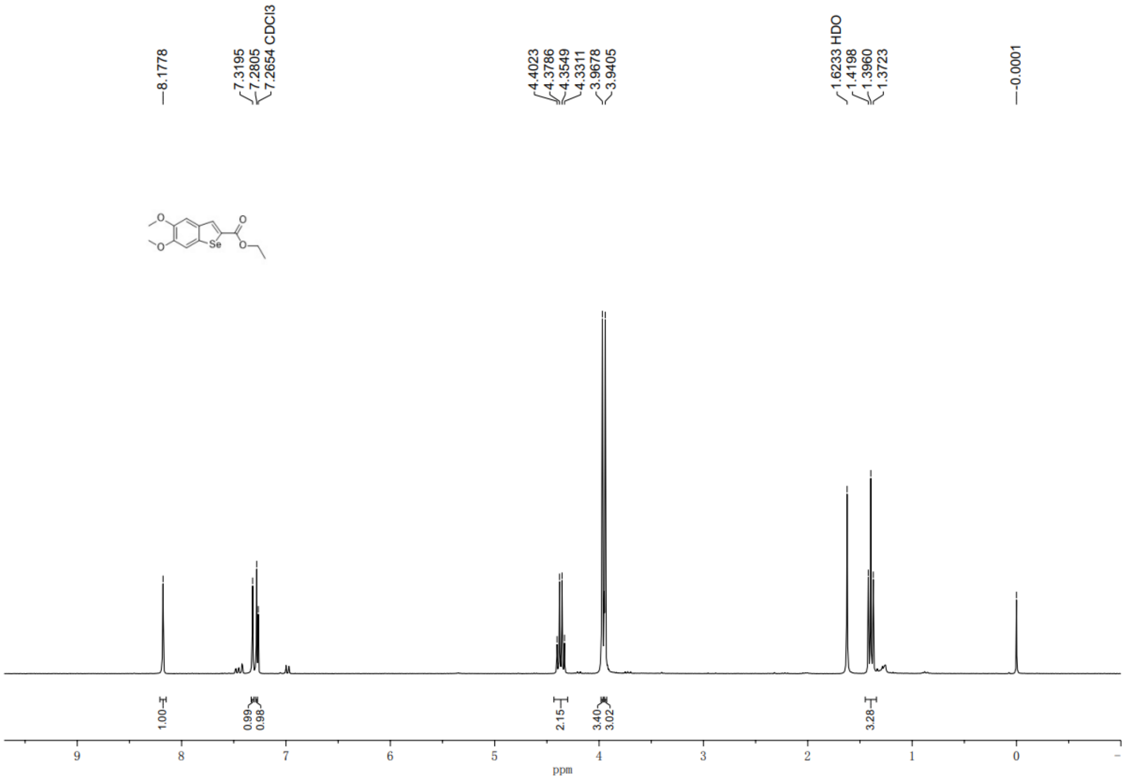

$^{1}$H NMR of compound **4** (300 MHz, $CDCl_3$)

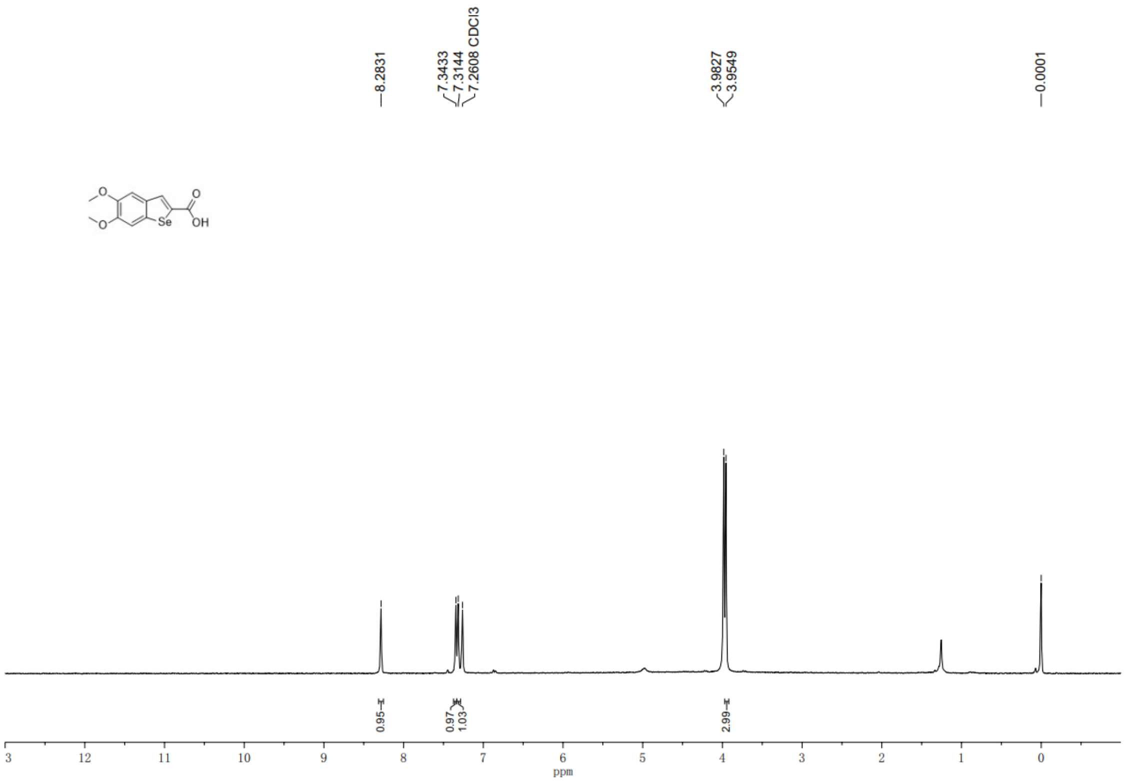


$^{1}$H NMR of compound **5** (300 MHz, $CDCl_3$)

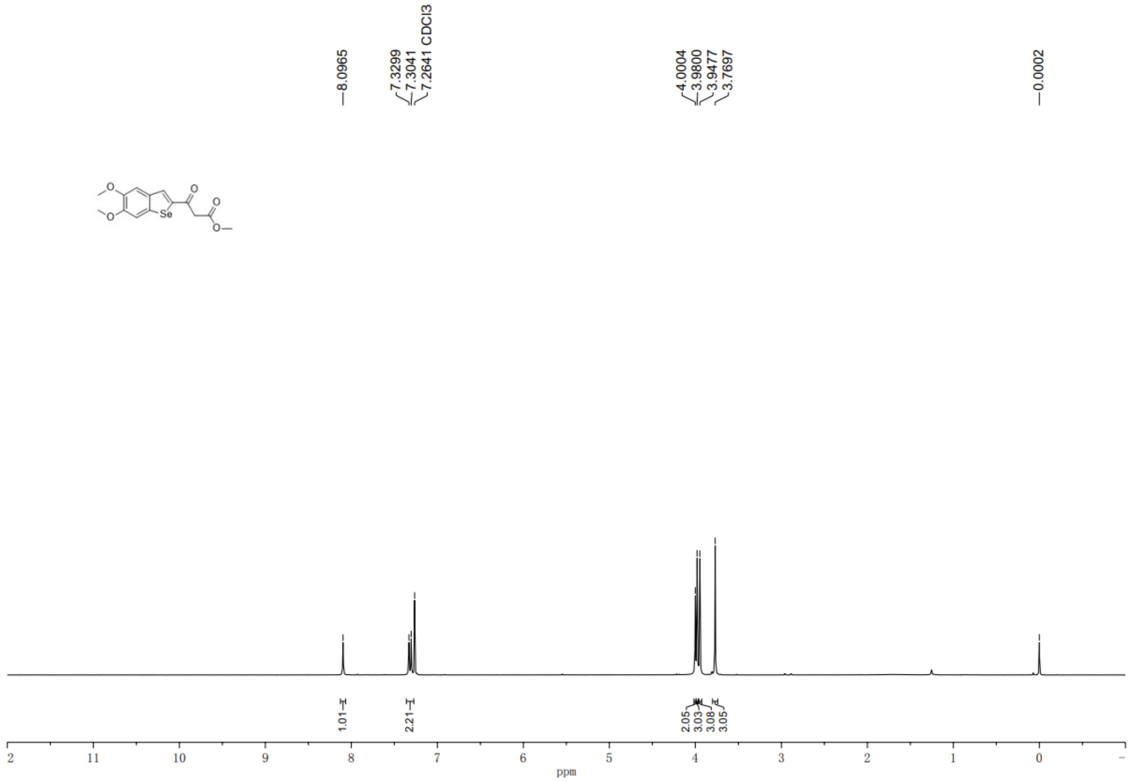


$^{1}$H NMR of compound **6** (300 MHz, $CDCl_3$)

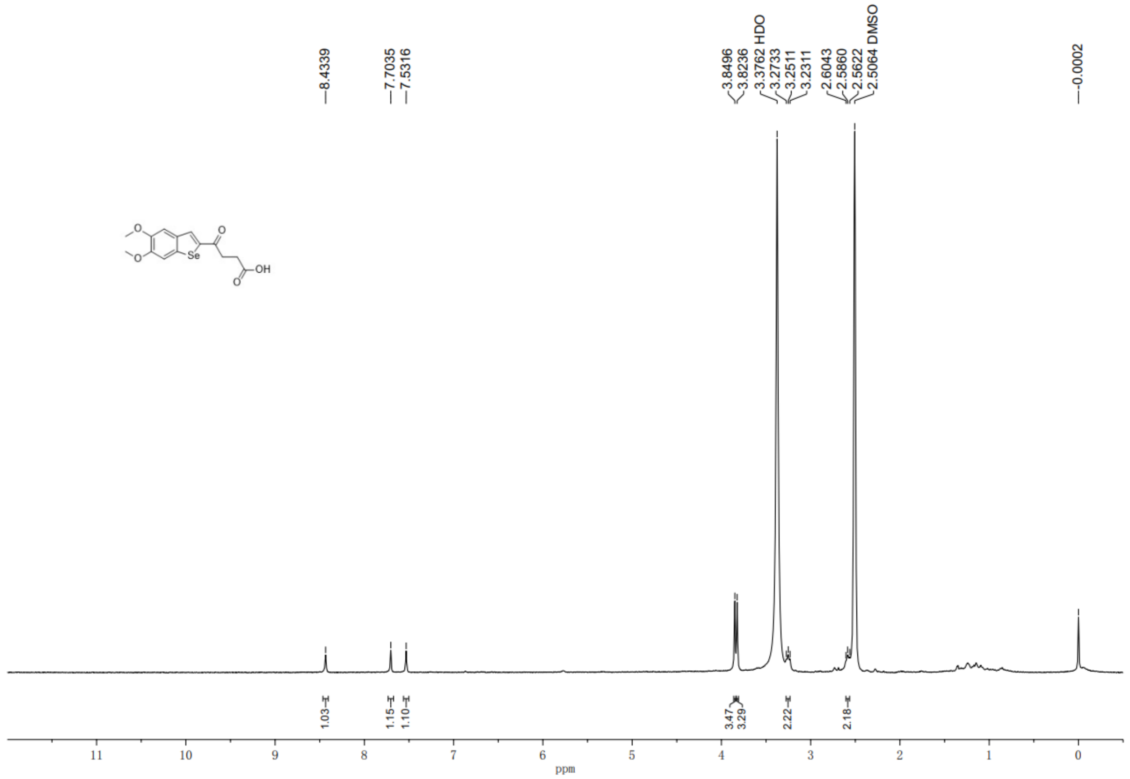

$^{1}$H NMR of compound **BSP01** (300 MHz, DMSO-$d_6$)

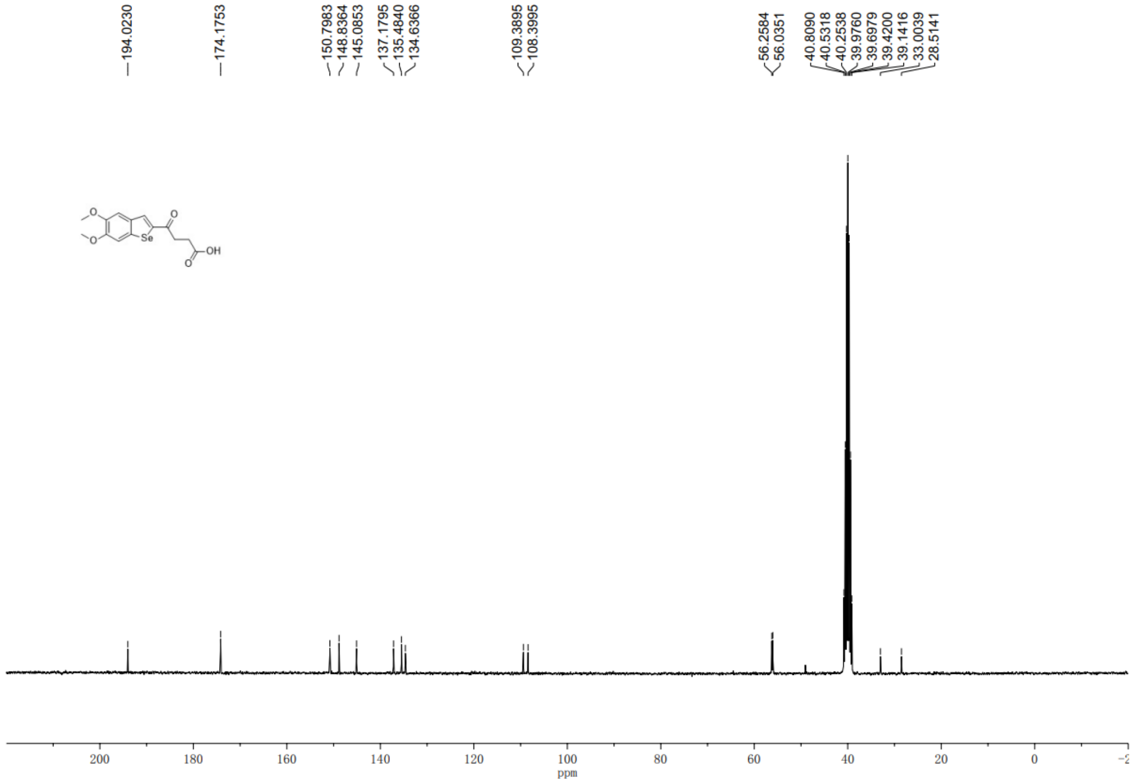

$^{13}$C NMR of compound **BSP01** (75 MHz, DMSO-$d_6$)

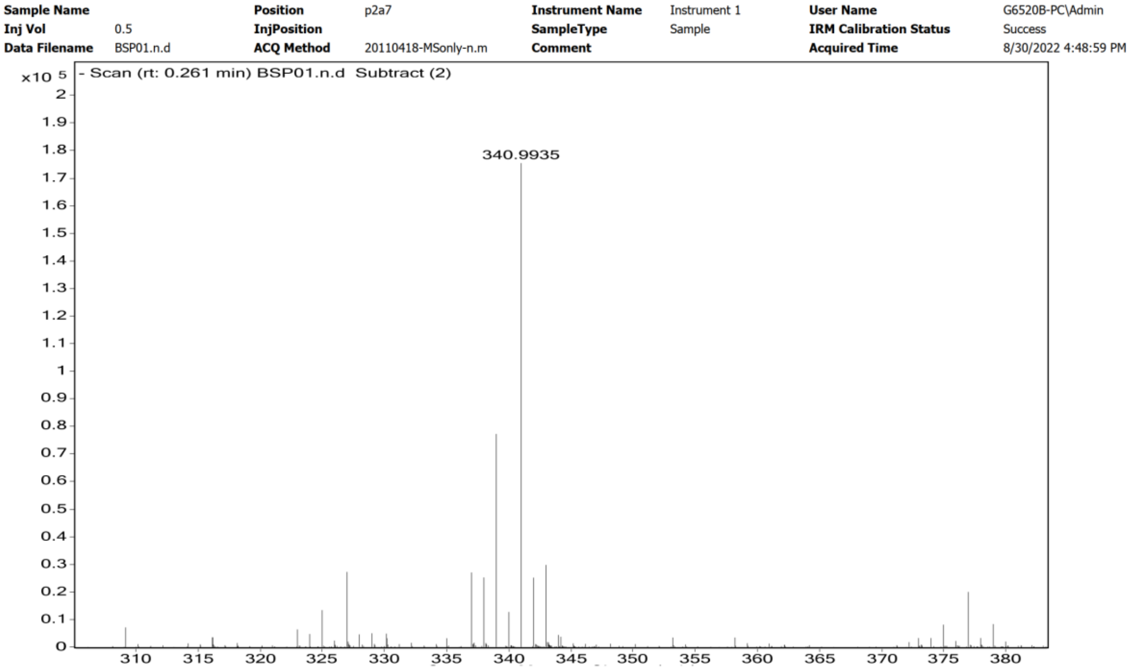


HRMS spectrum of compound **BSP01**

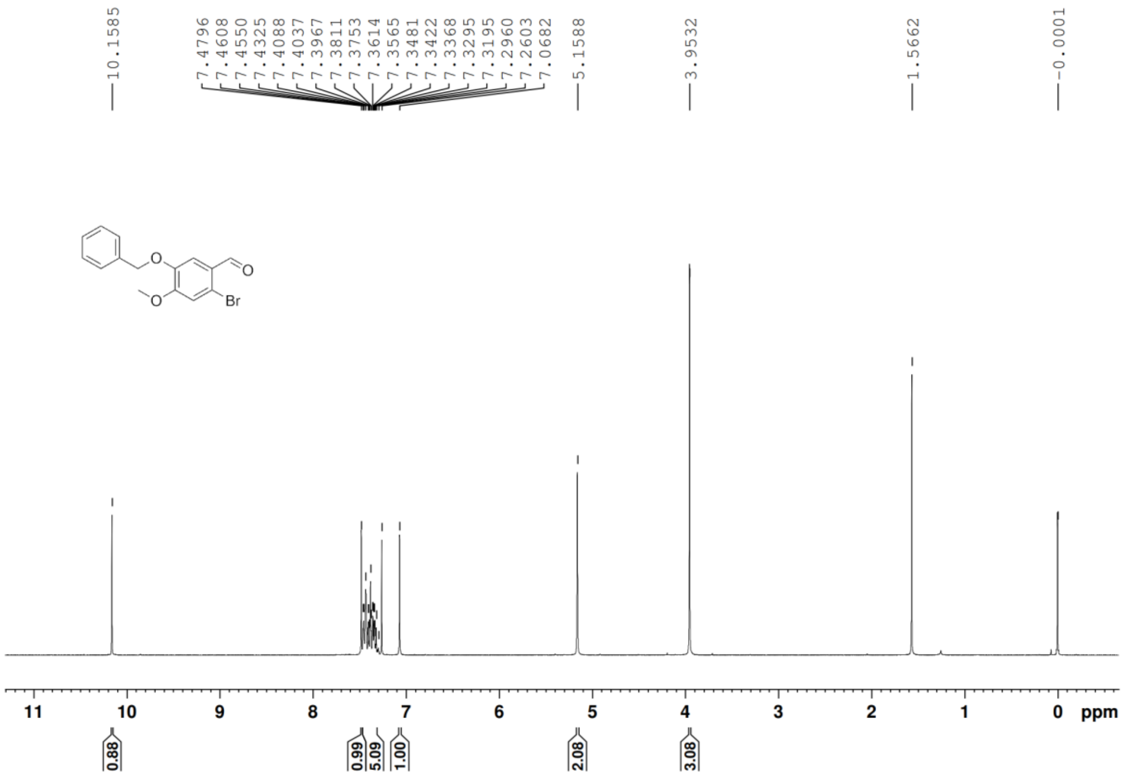


$^1$H NMR of compound **9** (300 MHz, $CDCl_3$)

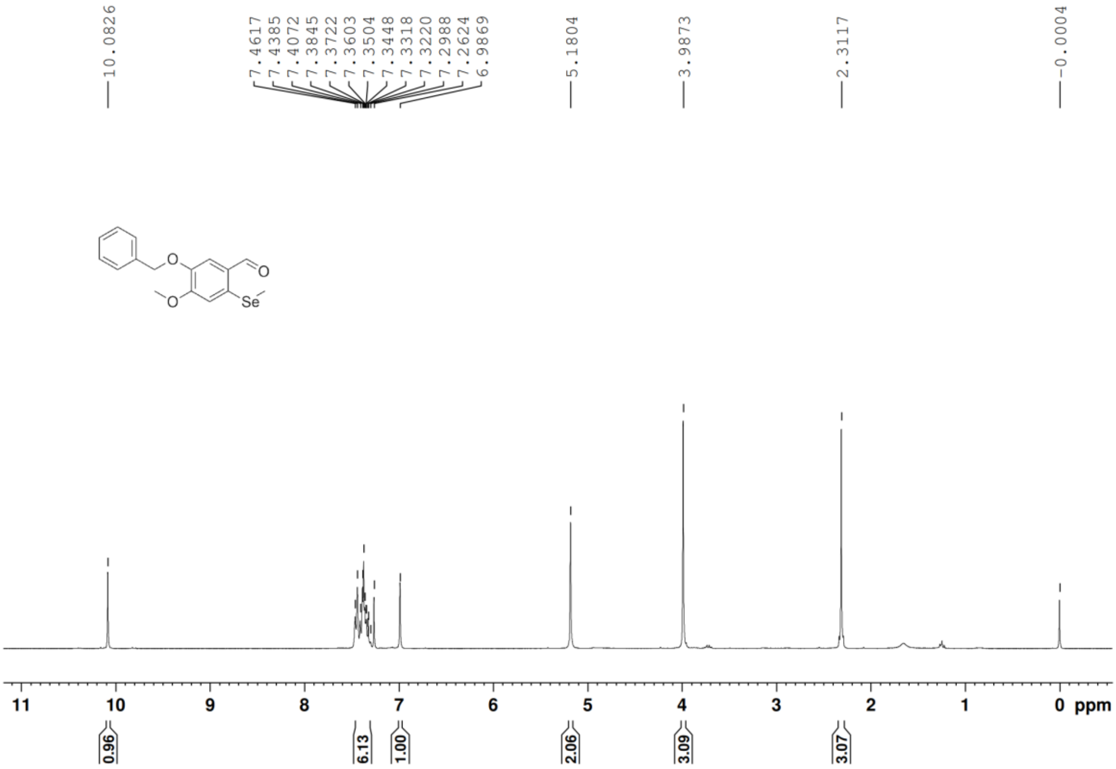


$^{1}$H NMR of compound **10** (300 MHz, $CDCl_3$)

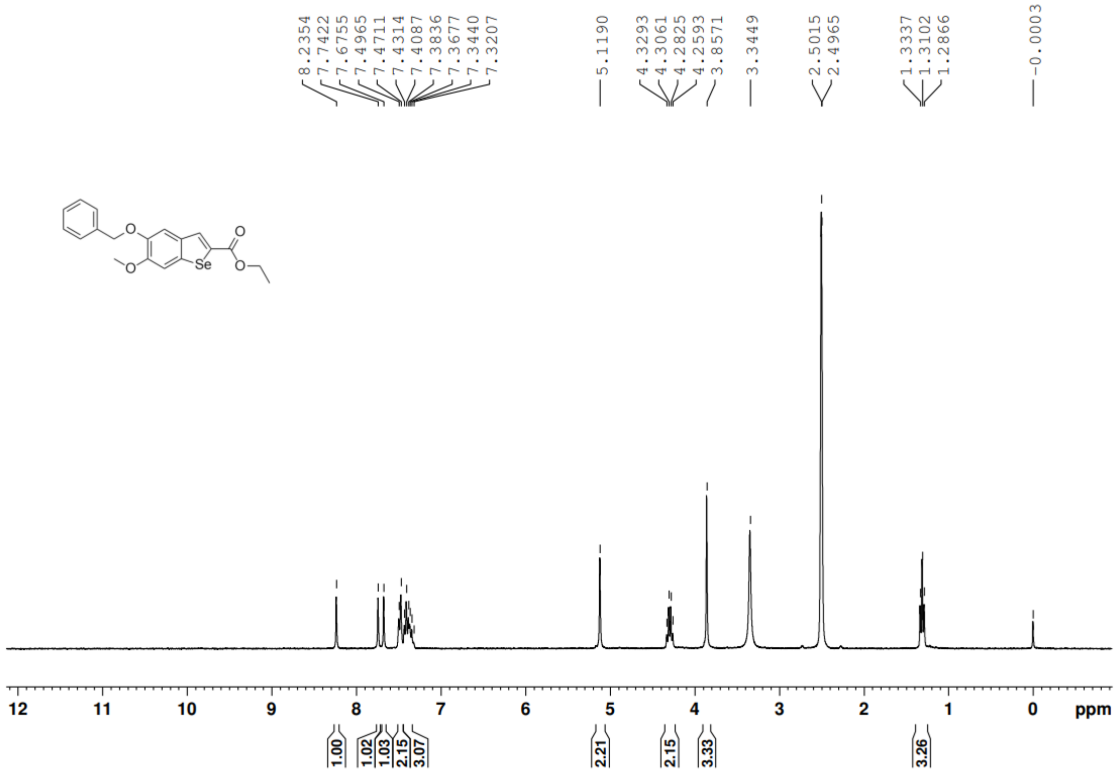


$^{1}$H NMR of compound **12** (300 MHz, DMSO-$d_6$)

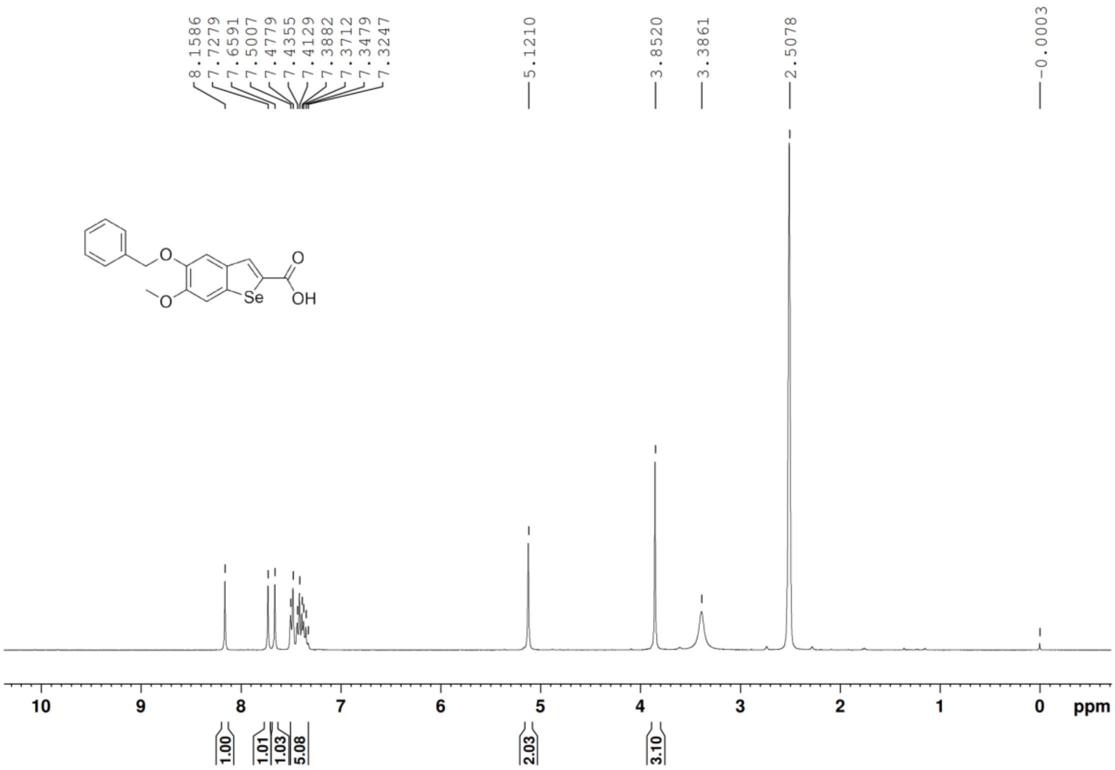


$^{1}$H NMR of compound **13** (300 MHz, DMSO-$d_6$)

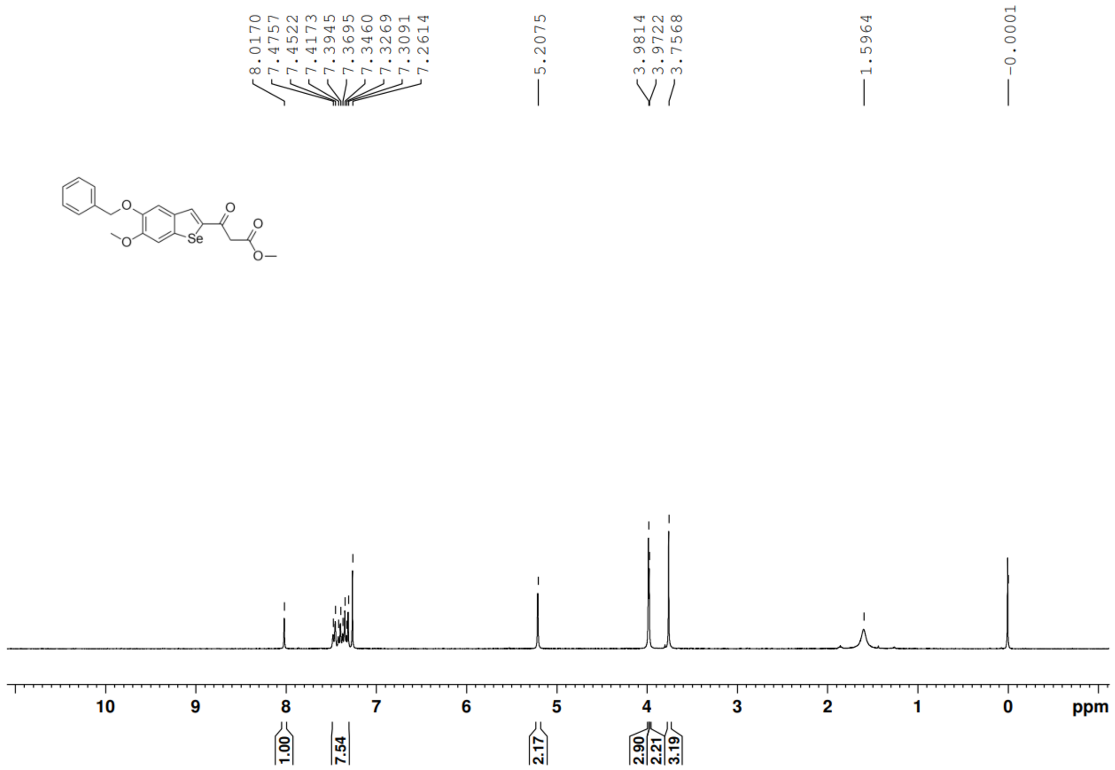


$^{1}$H NMR of compound **14** (300 MHz, $CDCl_3$)

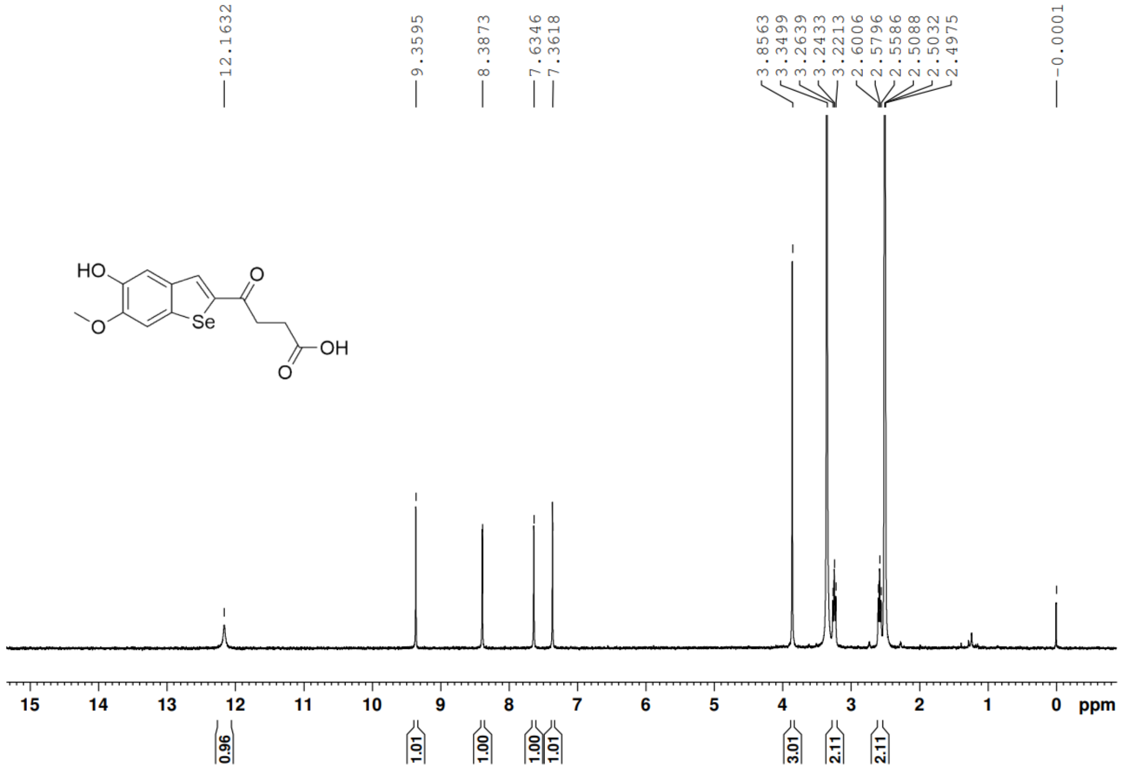


$^{1}$H NMR of compound **16** (300 MHz, DMSO-$d_6$)

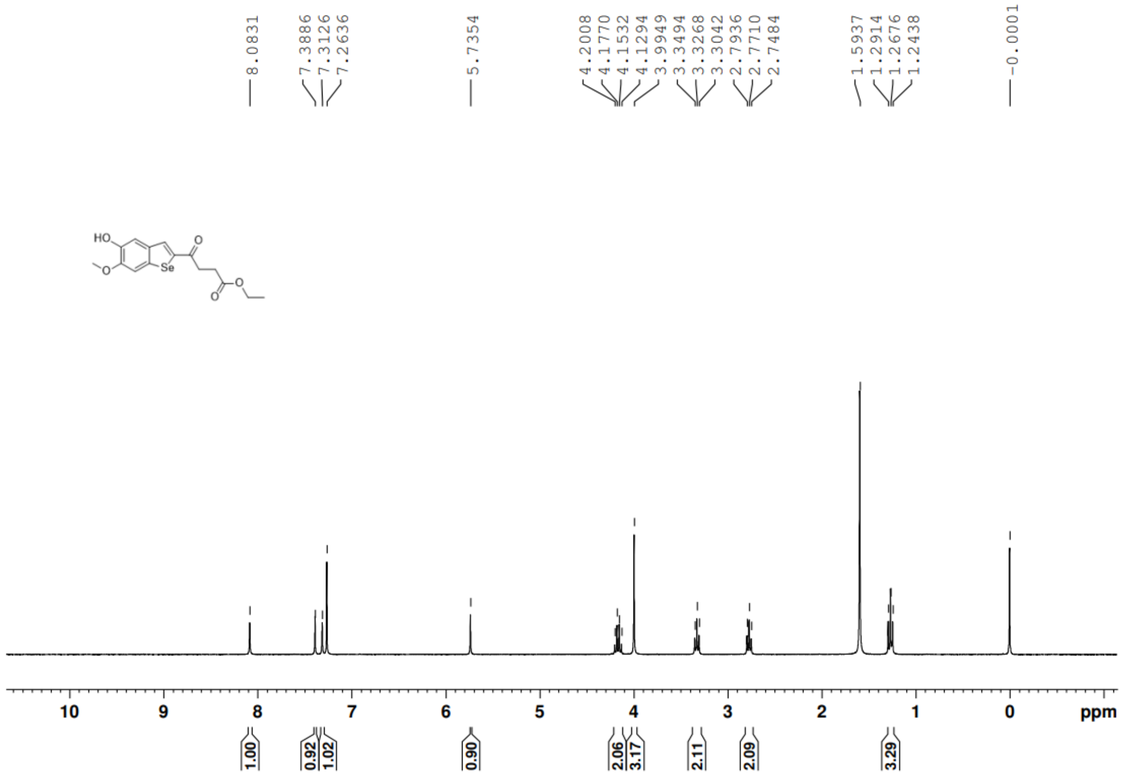


$^{1}$H NMR of compound **17** (300 MHz, $CDCl_3$)

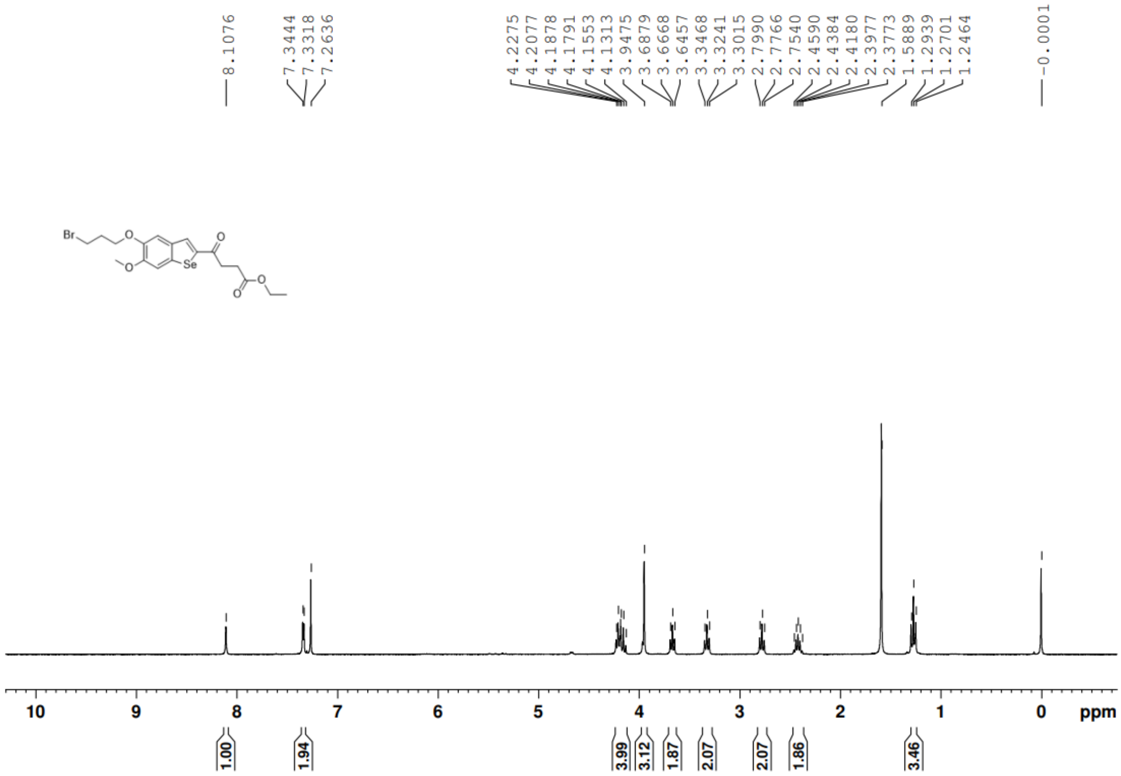


$^{1}$H NMR of compound **18** (300 MHz, $CDCl_3$)

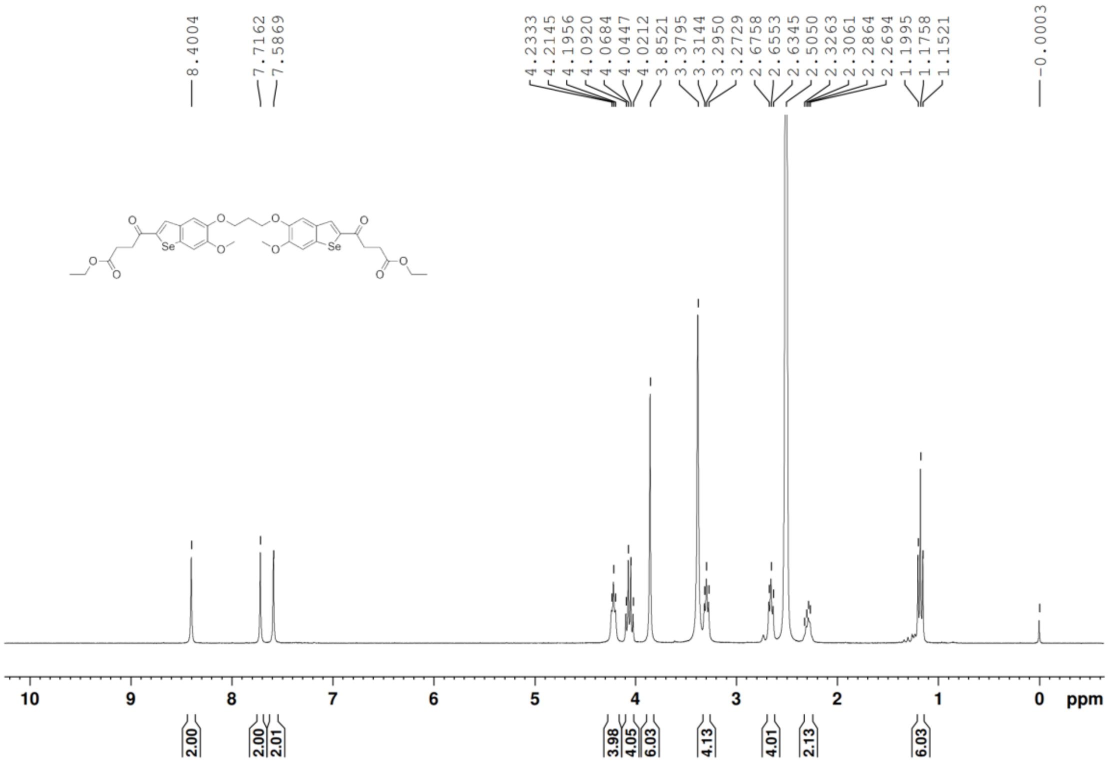


$^{1}$H NMR of compound **19** (300 MHz, DMSO-$d_6$)

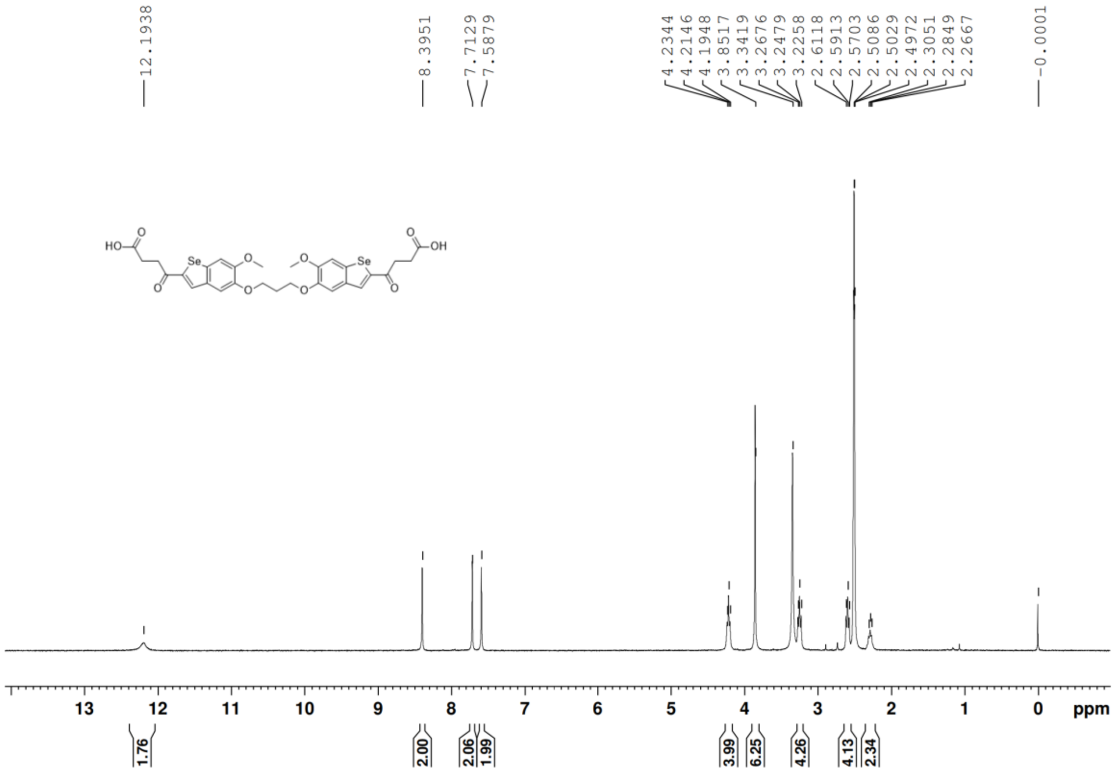


$^{1}H$ NMR of compound **diBSP01** (300 MHz, DMSO-$d_6$)

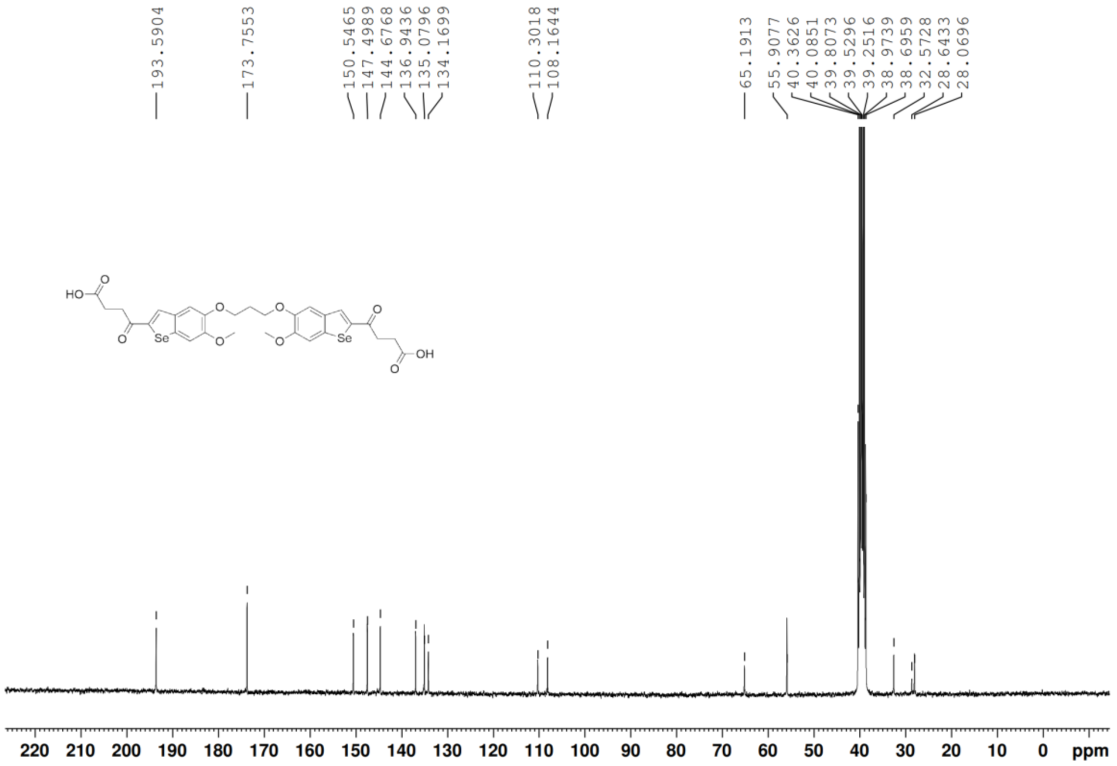


$^{13}C$ NMR of compound **diBSP01** (75 MHz, DMSO-$d_6$)

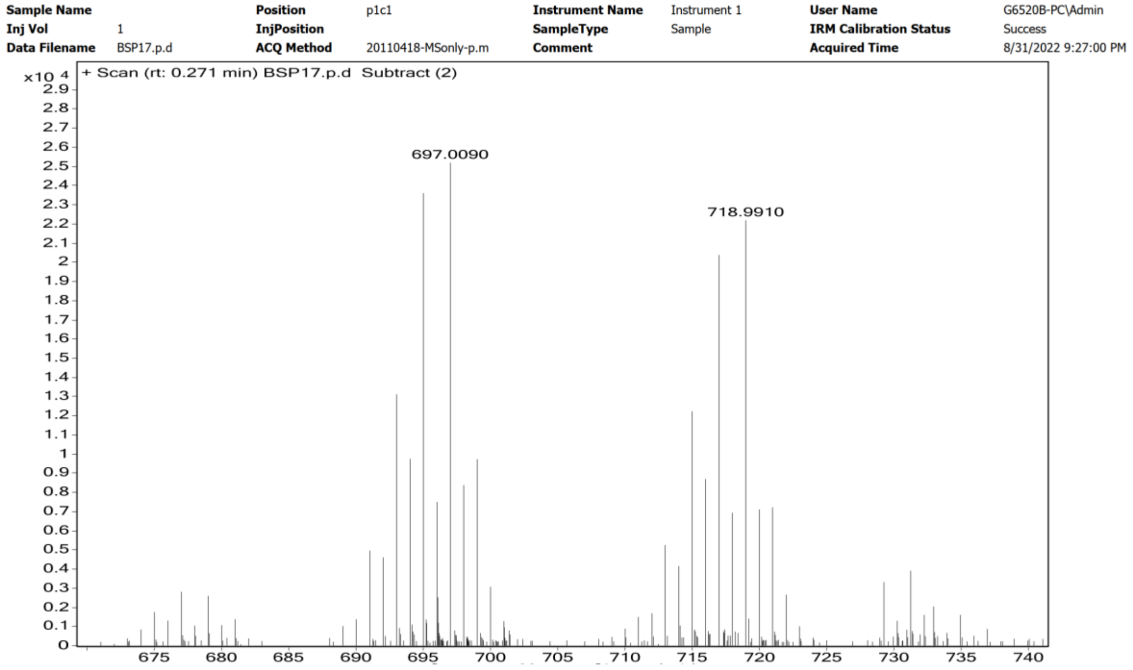


HRMS spectrum of compound **diBSP01**

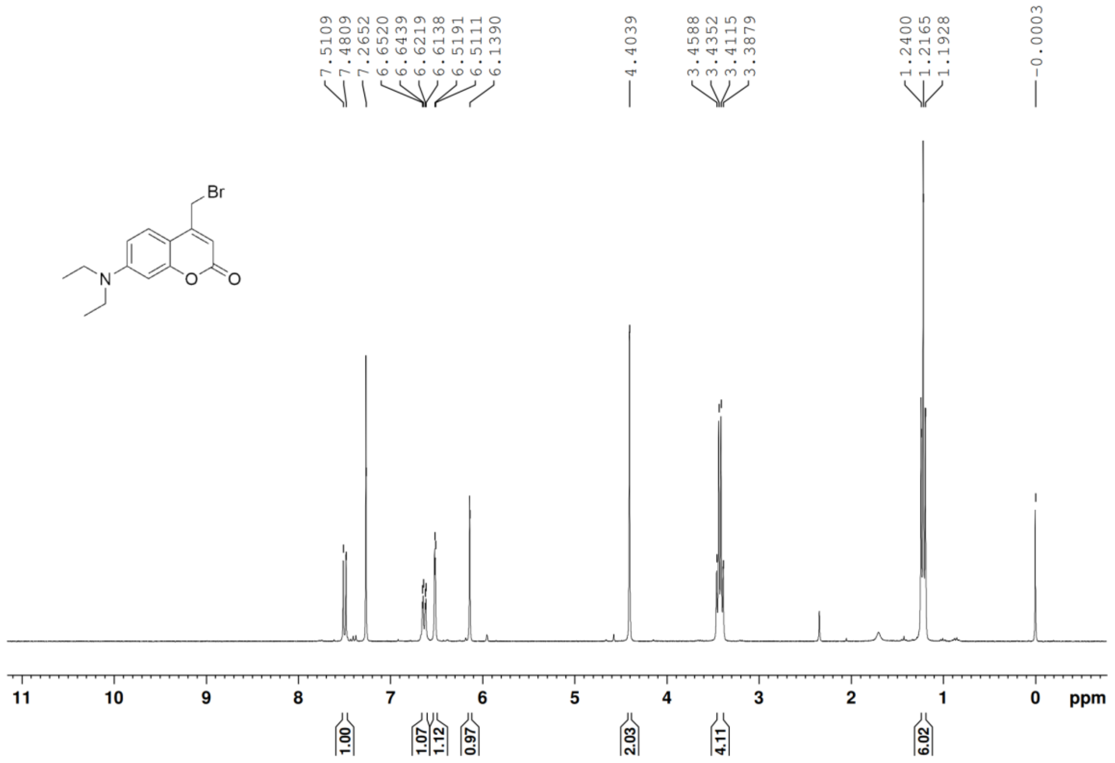


$^{1}$H NMR of compound **21** (300 MHz, $CDCl_3$)

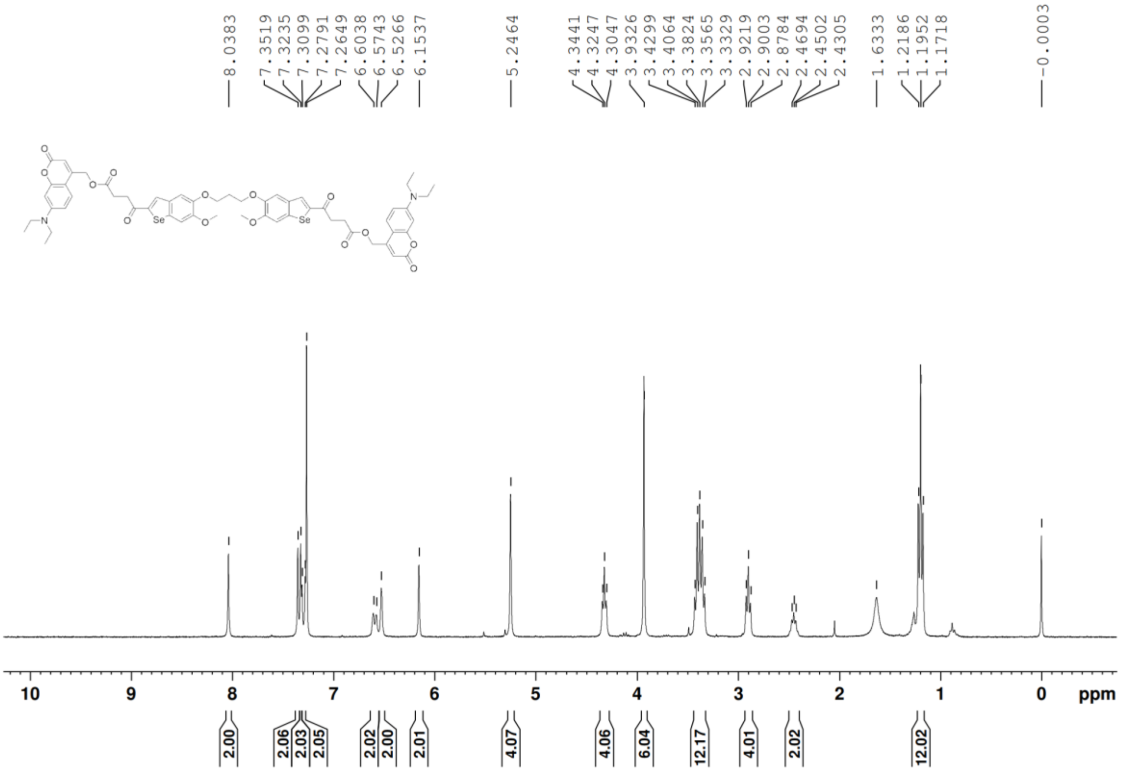


$^1$H NMR of compound caged-**diBSP01** (300 MHz, $CDCl_3$)

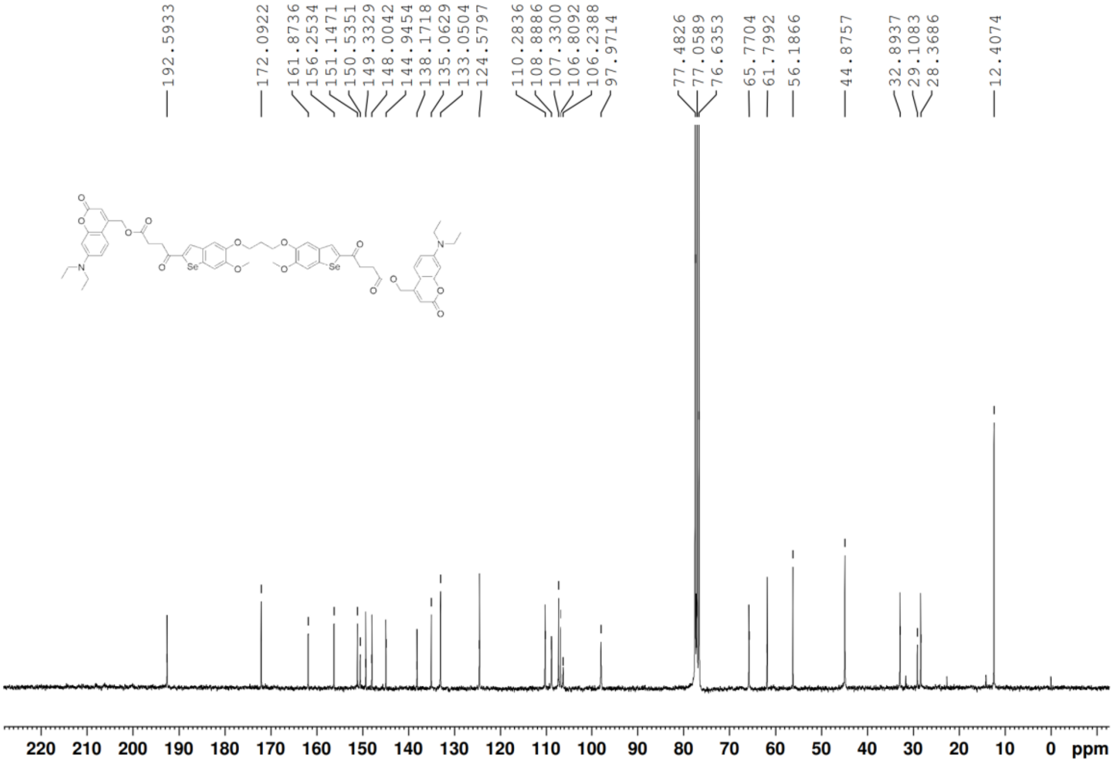


$^{13}$C NMR of compound caged-**diBSP01** (75 MHz, $CDCl_3$)

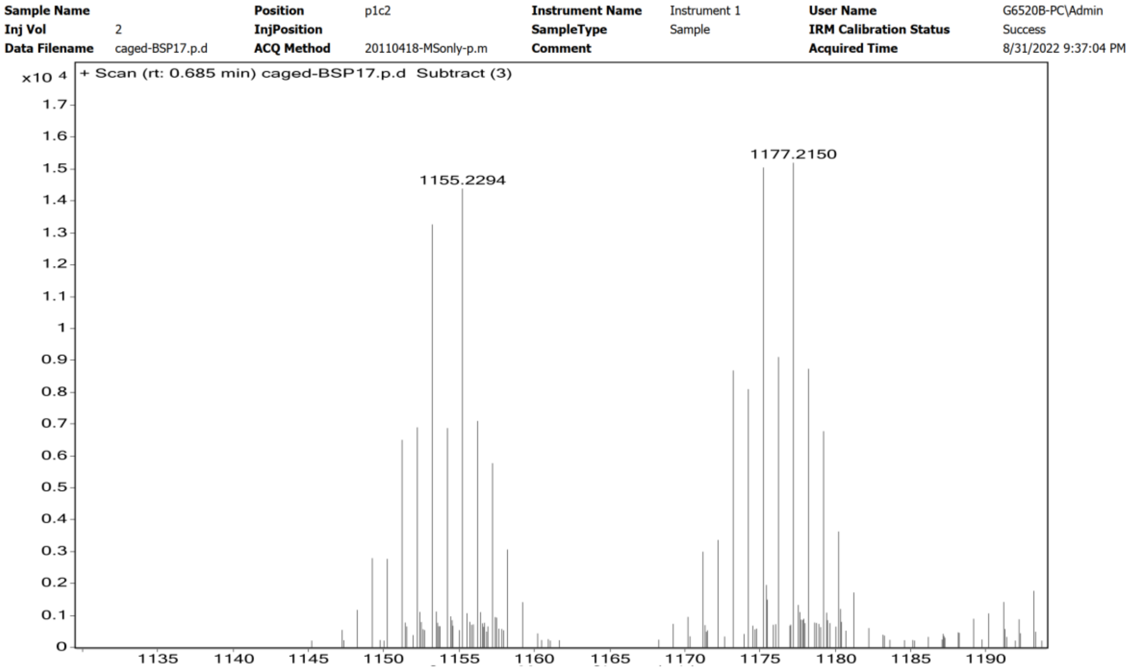


HRMS spectrum of compound caged-**diBSP01**

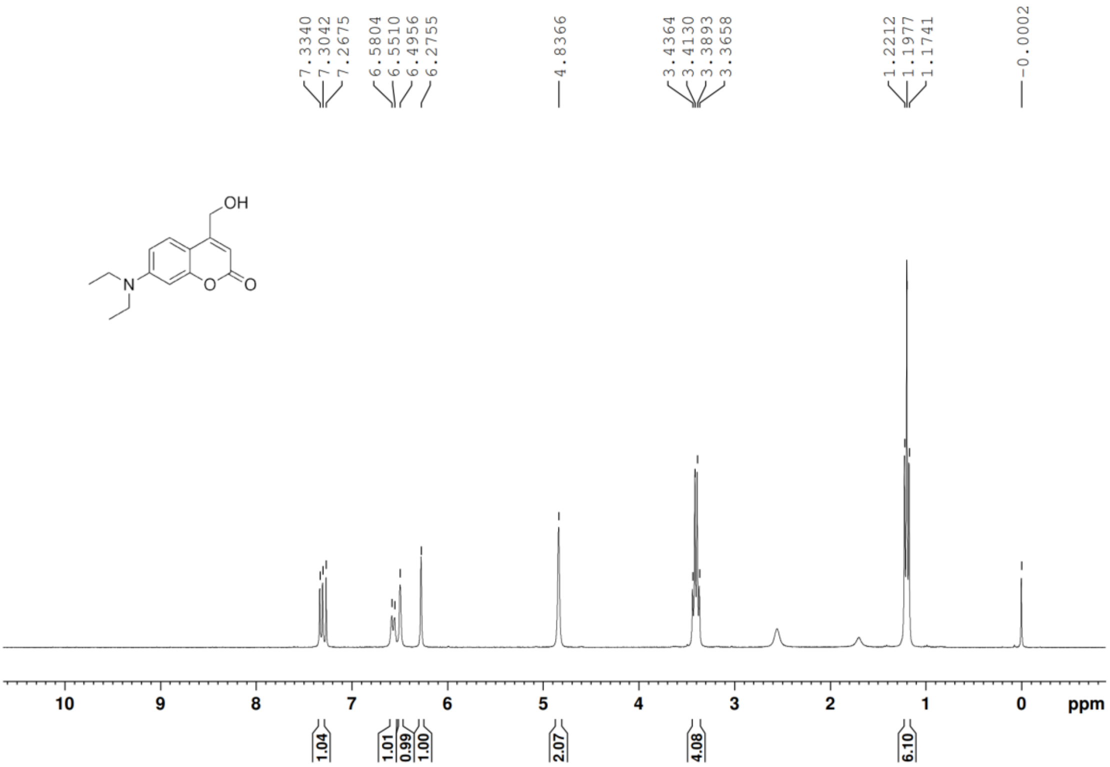


$^{1}$H NMR of compound PPG (300 MHz, $CDCl_3$)

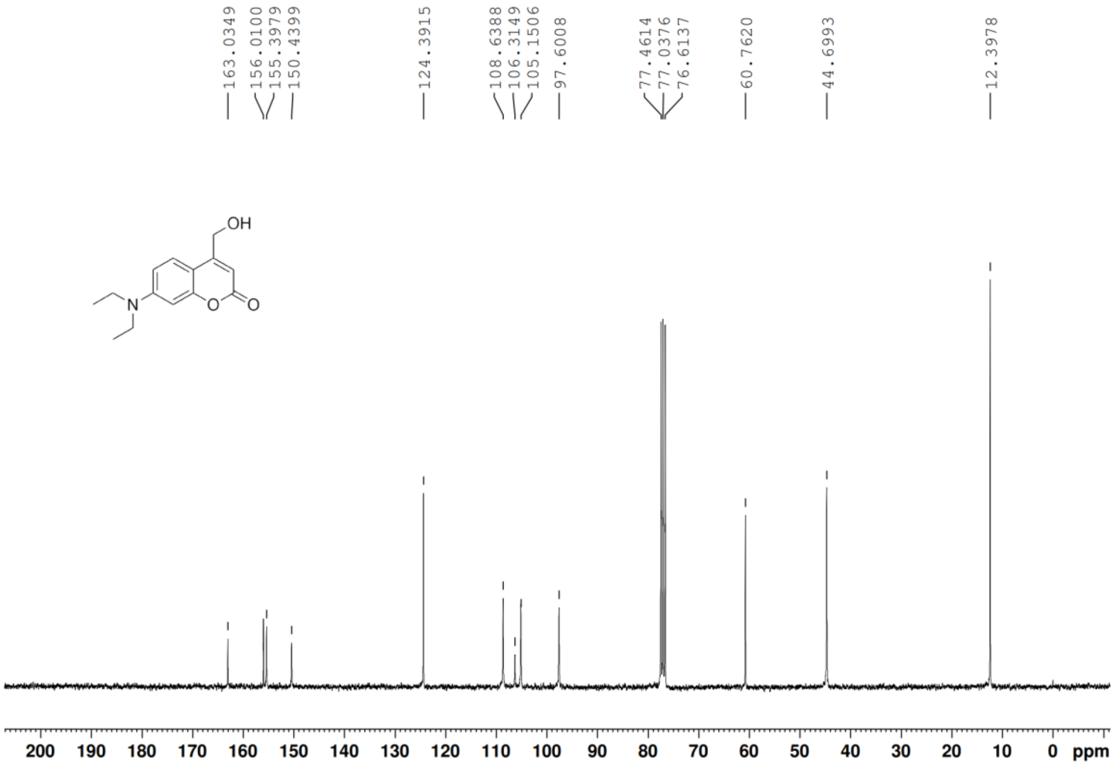


$^{13}C$ NMR of compound PPG (75 MHz, $CDCl_3$)

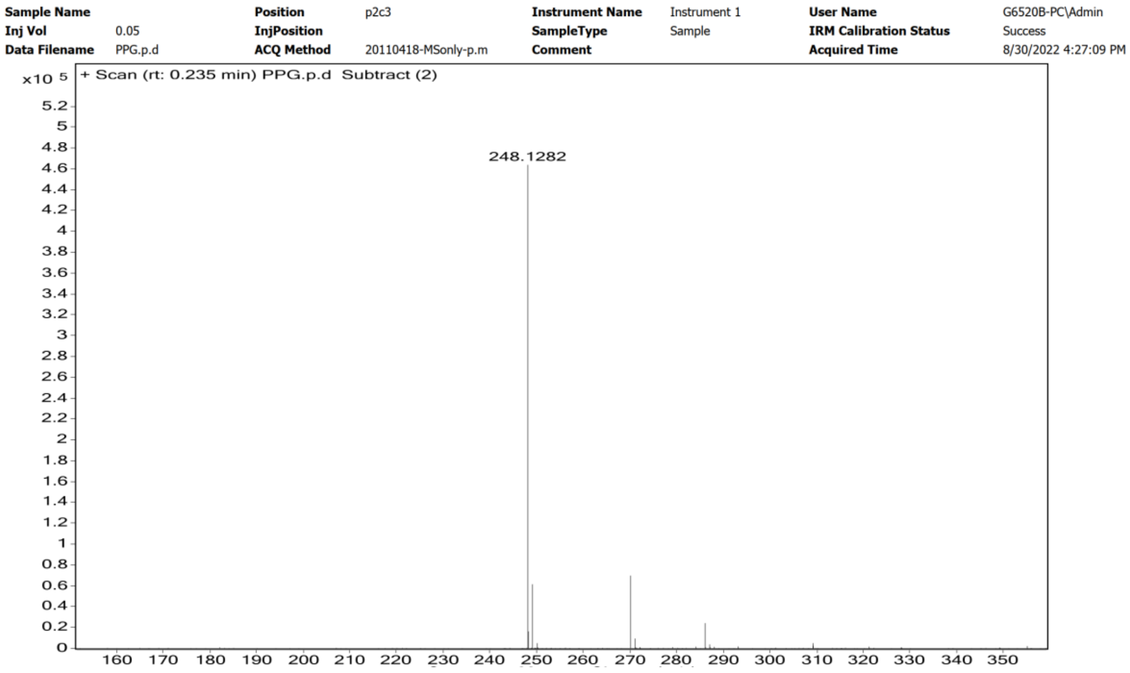


HRMS spectrum of compound PPG

# HPLC spectra of final compounds

Column: Hedera ODS-2 5 μm 4.6 × 250 mm

Mobile phase A: 0.1% formic acid/water

Mobile phase B: methanol

Gradient: 30−95% B over 10 min followed by a 5 min hold at 95% B, then a 5 min hold at 30% B

Wavelength: 254 nm

Flow rate: 1.0 mL/min

Temperature: 25 °C

Compound **BSP01** (99.4%)

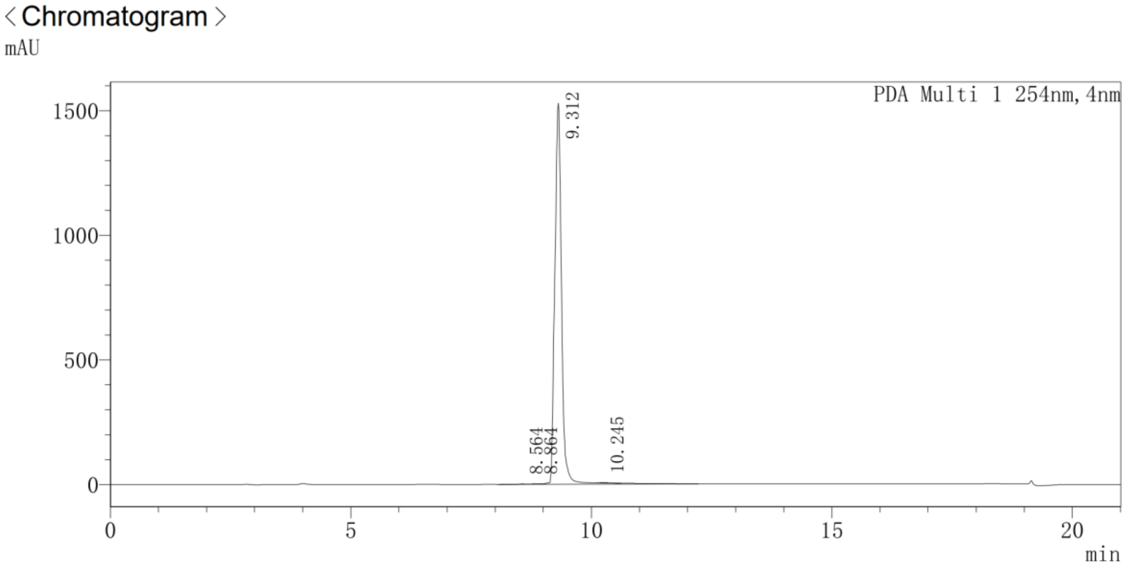


<Peak table>

PDA Ch1 254nm

| No. | R.T. | Area | Area% |
|---|---|---|---|
| 1 | 8.564 | 21566 | 0.144 |
| 2 | 8.864 | 35582 | 0.237 |
| 3 | 9.312 | 14939286 | 99.426 |
| 4 | 10.245 | 29068 | 0.193 |
| | | 15025502 | 100.000 |

Compound **diBSP01** (97.3%)

< Chromatogram >

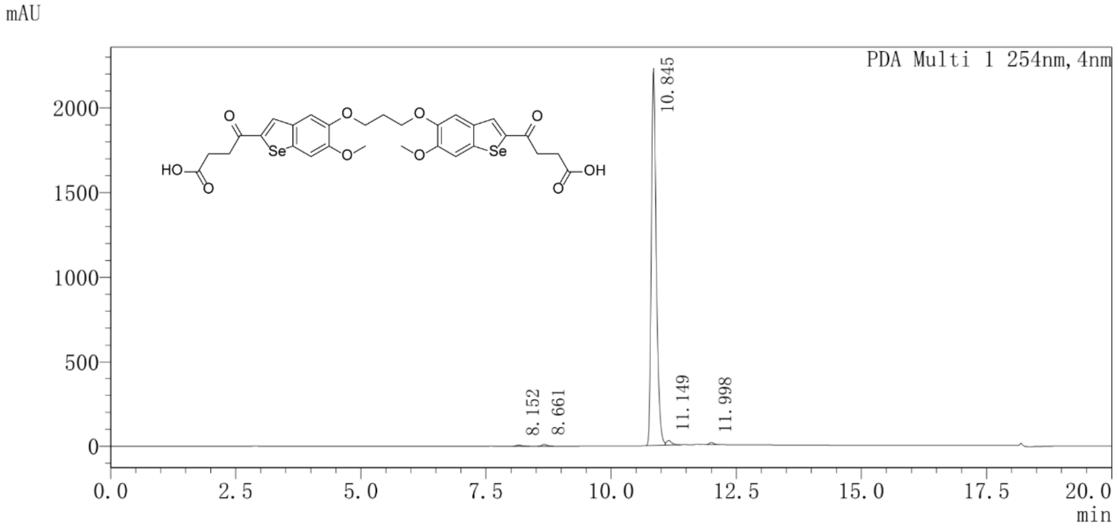


< Peak table >

PDA Ch1 254nm

| No. | R.T. | Area | Area% |
|---|---|---|---|
| 1 | 8.152 | 48855 | 0.314 |
| 2 | 8.661 | 71598 | 0.460 |
| 3 | 10.845 | 15159032 | 97.355 |
| 4 | 11.149 | 208483 | 1.339 |
| 5 | 11.998 | 82916 | 0.533 |
| Total | | 15570884 | 100.000 |

Compound caged-**diBSP01** (98.7%)

< Chromatogram >

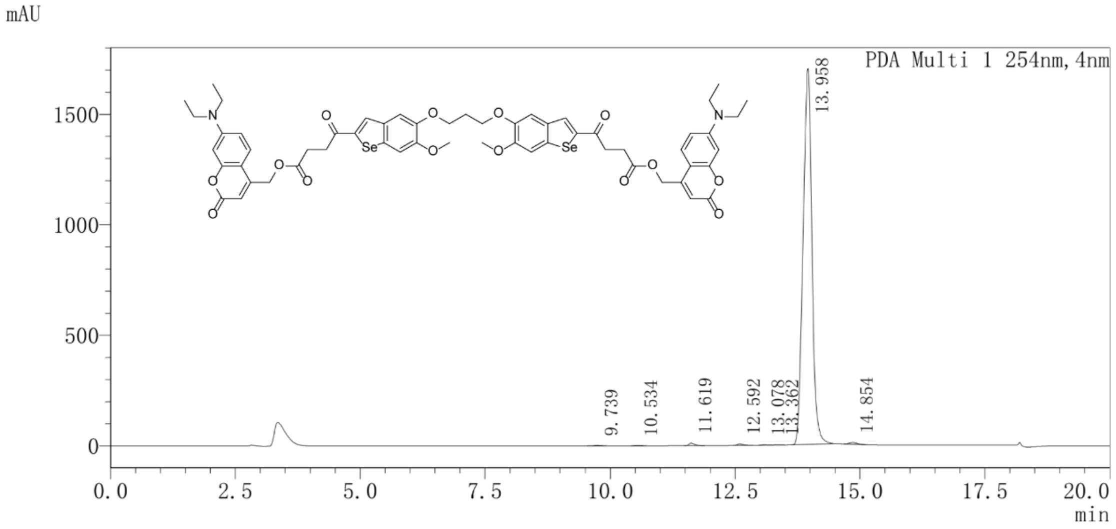


< Peak table >

PDA Ch1 254nm

| No. | R.T. | Area | Area% |
|---|---|---|---|
| 1 | 9.739 | 20124 | 0.095 |
| 2 | 10.534 | 8144 | 0.038 |
| 3 | 11.619 | 86112 | 0.405 |
| 4 | 12.592 | 52349 | 0.246 |
| 5 | 13.078 | 12699 | 0.060 |
| 6 | 13.362 | 12351 | 0.058 |
| 7 | 13.958 | 20971566 | 98.708 |
| 8 | 14.854 | 82810 | 0.390 |
| Total | | 21246156 | 100.000 |

Compound PPG (99.4%)

〈 Chromatogram 〉

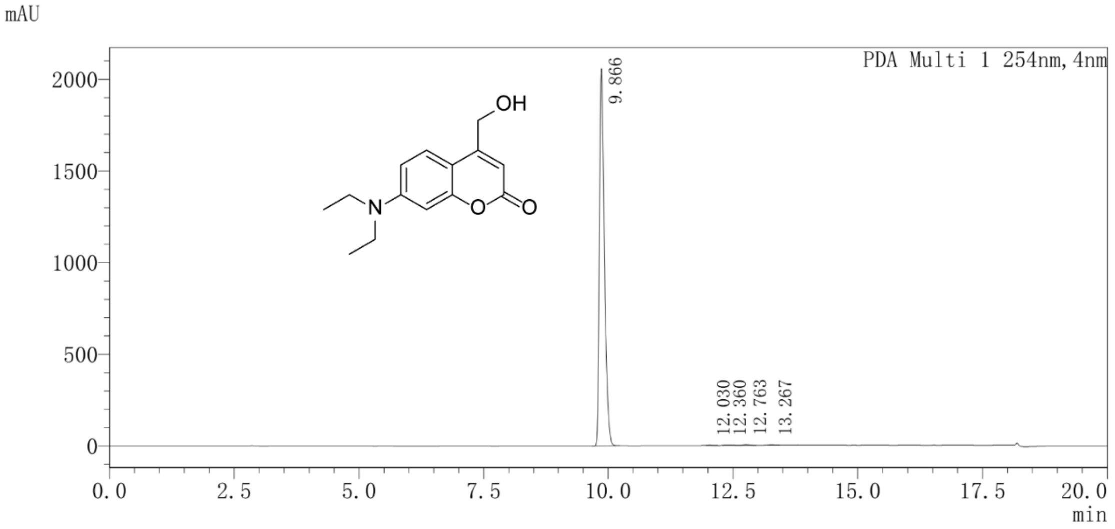


〈 Peak table 〉

PDA Ch1 254nm

| No. | R.T. | Area | Area% |
|---|---|---|---|
| 1 | 9.866 | 14196692 | 99.490 |
| 2 | 12.030 | 10822 | 0.076 |
| 3 | 12.360 | 21528 | 0.151 |
| 4 | 12.763 | 27336 | 0.192 |
| 5 | 13.267 | 13126 | 0.092 |
| Total | | 14269504 | 100.000 |

# References


1. R. Dubey, H. Lee, D.-H. Nam and D. Lim, *Tetrahedron Letters*, 2011, **52**, 6839-6842.
2. A. B. Mhetre, H. Lee, H. Yang, K. Lee, D. H. Nam and D. Lim, *Org Biomol Chem*, 2017, **15**, 1198-1208.
3. Z. Li, K. Su, Z. Jiang, Y. Yu, Q. You and X. Zhang, *J Med Chem*, 2019, **62**, 7583-7588.
4. F. Yu, Q. Wang, M. Li, H. Zhou, K. Liu, K. Zhang, Z. Wang, Q. Xu, C. Xu, Q. Pan and J. He, *Journal of Applied Crystallography*, 2019, **52**, 472-477.
5. D. Liebschner, P. V. Afonine, M. L. Baker, G. Bunkoczi, V. B. Chen, T. I. Croll, B. Hintze, L. W. Hung, S. Jain, A. J. McCoy, N. W. Moriarty, R. D. Oeffner, B. K. Poon, M. G. Prisant, R. J. Read, J. S. Richardson, D. C. Richardson, M. D. Sammito, O. V. Sobolev, D. H. Stockwell, T. C. Terwilliger, A. G. Urzhumtsev, L. L. Videau, C. J. Williams and P. D. Adams, *Acta Crystallogr D Struct Biol*, 2019, **75**, 861-877.
6. P. Emsley, B. Lohkamp, W. G. Scott and K. Cowtan, *Acta Crystallogr D Biol Crystallogr*, 2010, **66**, 486-501.
